# Integrated proteomics and metabolomics analysis of D-pinitol function during hippocampal damage in streptozocin-induced aging-accelerated mice

**DOI:** 10.3389/fnmol.2023.1251513

**Published:** 2023-10-30

**Authors:** Xiaoxia Li, Yuan Gao, Baoying Li, Wenqian Zhao, Qian Cai, Wenbin Yin, Shudong Zeng, Xiaoli Li, Haiqing Gao, Mei Cheng

**Affiliations:** ^1^Department of Geriatric Medicine, Qilu Hospital, Cheeloo College of Medicine, Shandong University, Jinan, China; ^2^Key Laboratory of Cardiovascular Proteomics of Shandong Province, Qilu Hospital, Cheeloo College of Medicine, Shandong University, Jinan, China; ^3^Jinan Clinical Research Center for Geriatric Medicine, Jinan, China; ^4^Department of Diabetes, The Third People's Hospital of Gansu Province, Lanzhou, China; ^5^Health Management Center (East Area), Qilu Hospital of Shandong University, Jinan, China; ^6^Department of Pharmacy, Qilu Hospital, Cheeloo College of Medicine, Shandong University, Jinan, China

**Keywords:** diabetes, hippocampal damage, D-pinitol, proteomics, metabolomics

## Abstract

**Purpose:**

Diabetes can cause hippocampal damage and lead to cognitive impairment. Diabetic cognitive impairment (DCI) is a chronic complication of diabetes associated with a high disability rate; however, its pathogenesis and therapeutic targets are unclear. We aimed to explore the mechanism of hippocampal damage during diabetes and evaluate the potential role of D-pinitol (DP) in protecting hippocampal tissue and improving cognitive dysfunction.

**Methods:**

DP (150 mg/kg/day) was administered intragastrically to streptozocin-induced aging-accelerated mice for 8 weeks. Hippocampal tissues were examined using tandem mass tag (TMT)-based proteomics and liquid chromatography-mass spectrometry (LC–MS)/MS-based non-targeted metabolomic analysis. Differentially expressed proteins (DEPs) and differentially regulated metabolites (DRMs) were screened for further analysis, and some DEPs were verified using western blotting.

**Results:**

Our results showed that 329 proteins had significantly altered hippocampal expression in untreated diabetic mice (DM), which was restored to normal after DP treatment in 72 cases. In total, 207 DRMs were identified in the DM group, and the expression of 32 DRMs was restored to normal post-DP treatment. These proteins and metabolites are involved in metabolic pathways (purine metabolism, arginine and proline metabolism, and histidine metabolism), actin cytoskeleton regulation, oxidative phosphorylation, and Rap1-mediated signaling.

**Conclusions:**

Our study may help to better understand the mechanism of diabetic hippocampal damage and cognitive impairment and suggest a potential therapeutic target.

## 1. Introduction

Diabetes is an endocrine and metabolic disease caused by insufficient insulin secretion or insulin resistance, which can be accompanied by diabetic angiopathy, neuropathy, diabetic cognitive impairment (DCI), and other complications (Zheng et al., 2018; Cuevas et al., 2023). There is growing evidence to support the hypothesis that patients with diabetes exhibit hippocampal damage and DCI (den Heijer et al., 2003; Hayashi et al., 2011). In these patients, hippocampal neurons undergo degeneration, necrosis, apoptosis, and other pathological changes due to hyperglycemia, resulting in hippocampal damage. Therefore, the pathophysiological characteristics of hippocampal damage need to be further studied.

The hippocampus, located in the cerebral cortex, is a brain region responsible for learning and memory formation and storage over a long time. At present, many pathogenetic mechanisms underlying hippocampal damage and DCI have been discovered, including insulin resistance, neuroinflammation, oxidative stress, advanced glycation end products, mitochondrial dysfunction, activation of hippocampal neuronal apoptosis, and ferroptosis signaling pathways (Dutta et al., 2022; Tang et al., 2022; Gupta et al., 2023). However, an effective treatment for hippocampal damage has not been proposed. D-Pinitol (DP) is a derivative of D-chiral inositol methylation and is a natural compound that originates from soybean and soybean products. It has been suggested that DP regulates islet function through insulin sensitization and mimicry (Azab, 2022). Many studies have shown that this compound has anti-inflammatory and antioxidant properties, protects the heart, and helps treat anxiety, depression, and convulsions (Alonso-Castro et al., 2019). However, the effects and molecular mechanisms of DP in the treatment of DCI, particularly during hippocampal damage, remain unclear.

Proteomics and metabolomics have been widely used in neuropsychology. Tandem mass tag (TMT) proteomics has shown increasing value for high-throughput and high-precision protein detection and analysis (Sandberg et al., 2014). Therefore, TMT technology is an ideal method for comprehensively analyzing the effect of DP on protein expression in the hippocampus of diabetic mice and its underlying mechanism. Metabolomics is an emerging technology that utilizes high-throughput techniques such as mass spectrometry to study the metabolites within an organism. Liquid chromatography-mass spectrometry (LC–MS)-based metabolomics has high sensitivity, resolution, and a wide dynamic range. It has been used to decipher metabolic reprogramming in a variety of diseases, including neurodegenerative diseases (Shao et al., 2021). The discovery of differentially expressed proteins (DEPs) and differentially regulated metabolites (DRMs) is of great significance in revealing molecular alterations during DCI and hippocampal damage. Therefore, in this study, TMT proteomics and LC–MS-based metabolomics were adopted to investigate the molecular mechanism of hippocampal damage in a streptozotocin (STZ)-induced senescence-accelerated prone 8 (SAMP8) mouse model, with or without DP administration. In addition, this study aimed to investigate the potential targets of DP. Furthermore, western blotting was used to verify the differential proteins, which were analyzed using different bioinformatics tools.

## 2. Materials and methods

### 2.1. Materials

DP (purity: 95%, batch number: 441252) and STZ were purchased from Sigma–Aldrich (St. Louis, MO, USA). Acetonitrile and methyl alcohol were purchased from Millipore (Billerica, MA). TMT 10 plex kit was purchased from Thermo Fisher Scientific (Carlsbad, CA, USA). The antibodies for secernin, copine-6, septin-5, profilin 2, and β-actin were all purchased from Proteintech (Wuhan, China). All other chemical reagents used were of analytical grade.

### 2.2. Animals

Detailed information on the animal model is provided in the Supplementary material section. All experimental procedures were carried out in accordance with the animal program guidelines and regulations, and were approved by the Animal Protection and Use Committee of Shandong University (approval number: 21170). The mice in the four groups were the senescence-accelerated mouse resistant 1 (SAMR1) group (R1, *n* = 10), SAMP8 group (CC, *n* = 10), STZ-induced SAMP8 group (DM, *n* = 13), and DP-treated STZ-induced SAMP8 group (DP, 150 mg/kg, *n* = 13).

### 2.3. Estimation of body weight, fasting blood glucose (FBG), and behavioral tests

Mice were weighed at the end of 8 weeks, and FBG levels were measured using a DVI-1650 Automatic Biochemistry and Analysis instrument (Bayer, Germany). The Morris water maze test (MWM) apparatus was a round pool with a diameter of 120 cm, height of 60 cm, and water depth of 15 cm, and a circular platform with a diameter of 6 cm was placed in the fourth quadrant; the water was added to the pool until the water surface reached 1 cm above the platform, while the temperature was maintained at 21.5°C ± 0.5°C. MWM tests were performed as previously described in previous studies (Svensson et al., 2017; Bouter et al., 2018). Briefly, during the navigation experiment (days 1–4), each mouse was instructed to locate the concealed platform within 60 s. The search trajectory was recorded using a video camera, and software was used to measure the escape latency. A space probe trial with platform removal was performed on day 5. The number of target platform crossings was recorded. The SANS Animal Behavior Analysis System (Shanghai, China) was used to analyze the data.

The step-down test, a passive avoidance response test, was used in this study. The apparatus consisted of a model YLS-3 TB step-down recorder and five mouse step-down compartments of equal size (120 mm × 120 mm × 180 mm; Beijing, China). An insulated high platform was provided at the center of the compartment, and the bottom was paved with an electric copper mesh (3 mA). The experimental procedures were performed as previously described (Sakaguchi et al., 2003; Dimitrova and Getova-Spassova, 2006). The animals were tested in two trials (day 1 and day 2). During the testing period, the time of the first jump off the platform within 5 min was recorded as latency. The total number of times the mice jumped off the platform within 5 min was recorded as the number of errors.

### 2.4. Histopathology

The hippocampal tissues of three mice in each group (R1, CC, DM, and DP) were fixed with 4% formaldehyde, embedded in paraffin, and sectioned into coronal sections with a thickness of 3 μm. Then, HE and Nissl’s staining were applied.

### 2.5. TMT-based proteomics analysis

Hippocampal tissues of nine mice in each group (CC, DM, and DP) were selected for protein extraction. Three samples were pooled from each group. Briefly, the process of proteomics analysis is as follows: The hippocampal tissue was lysed using the SDT method, followed by quantification using the BCA method. According to the FASP procedure (Wisniewski et al., 2009), 300 μg of the protein from each sample was digested. The same number of peptides were taken from each sample and labeled according to the instructions of the Thermo Fisher TMT labeling kit. The labeled peptides in each group were mixed in the same amount, and the dried peptides were separated by Pierce reversed High pH Reversed-Phase Peptide Fractionation Kit (Thermo Fisher). Finally, the sample collection was merged into 10 components. The Q-Exactive HF-X mass spectrometer (Thermo Scientific) for mass spectrometry analysis. The original RAW file of LC–MS/MS is imported into the search engine Sequest HT in the Proteome Discoverer software (version 2.4, Thermo Scientific) for database retrieval. Differentially expressed proteins (DEPs) were screened by fold change (FC) ≥ 1.1 or ≤ 0.91 and *p* < 0.05 at the same time. The proteomic data are available from the Proteome Xchange Consortium (PXD043127).

### 2.6. Untargeted metabolomics analysis

The hippocampal tissues of nine mice from each group (CC, DM, and DP) were selected for metabolite extraction. Briefly, the process of metabolomics analysis is as follows: Samples were weighed and ground using a grinder. Metabolites were extracted using a pre-cooled mixture of methanol, acetonitrile, and water (in the ratio of 2:2:1, v/v/v), followed by 1 h of ultrasonic shaking in ice baths. Quality control (QC) samples were prepared by pooling representative aliquots of all samples and used for data normalization. The sample was separated by SHIMADZU-LC30 ultra-high-performance liquid chromatography (UHPLC) and HILIC column. Positive ion (+) and negative ion (−) patterns were detected by electrospray ionization (ESI) in each sample. After UPLC separation, the samples were analyzed by QEPlus mass spectrometer (Thermo Scientific) and ionized by HESI source. The original data were calibrated by MSDIAL software for peak alignment, retention time correction, and peak area extraction. Public databases such as HMDB and MassBank database were searched. Normalizing the total peak area of the positive and negative ion data, integrating the positive and negative ion peaks, and using R software for pattern recognition, the data were preprocessed by unit variance scaling (UV). Multivariate statistical analysis: R (version 4.0.3) was used for all multivariate data analyses and modeling. Data were mean-centered using Pareto scaling. Models were built on principal component analysis (PCA), orthogonal partial least-square discriminant analysis (PLS-DA), and partial least-square discriminant analysis (OPLS-DA). The selection of differential metabolites had both multivariate statistical analysis (VIP > 1) and univariate statistical analysis (*p* < 0.05 and FC > 1.5 or < 0.67).

### 2.7. Integrated analysis of proteomics and metabolomics

R 4.0.1 and Cytoscape 3.8.2 were used to analyze the significantly enriched pathways and molecular interaction networks of differentially expressed proteins and metabolites.

### 2.8. Western blot analysis

Hippocampal tissue proteins were extracted. Equal amounts of protein were separated using sodium dodecyl sulfate-polyacrylamide gel electrophoresis (SDS-PAGE) and then transferred to polyvinylidene fluoride (PVDF) membranes. The membranes were incubated at room temperature in a closed solution containing 5% skimmed dried milk for 1 h and then incubated with antibodies against secernin, copine-6, septin-5, profilin 2, and β-actin at 4°C overnight. The membranes were washed with TBS-T, incubated with an enzyme-conjugated secondary antibody, and visualized using an enhanced chemiluminescence kit (Thermo Pierce). β-actin was used as an internal reference. Finally, the images were exposed to ECL luminescence and X-ray film (Tanon, China) and analyzed using ImageJ 1.8 software.

### 2.9. Statistical analysis

The data are expressed as the mean ± standard deviation or standard error. The Student’s *t*-test was used to compare the two groups. The three groups of sample components were compared using a single-factor analysis of variance (ANOVA). The obtained LC–MS/MS raw files were searched using the Sequest HT engine embedded into Proteome Discoverer software (version 2.4, Thermo Scientific). Peptide false discovery rate FDR ≤0.01 GO and KEGG enrichment analyses were performed using Fisher’s exact test, and FDR correction for multiple testing was also performed. The significance level was set to *p* ≤ 0.05. The calculations were carried out using IBM SPSS 26.0 (Armonk, USA).

## 3. Results

### 3.1. Effects of the DP on body weight, FBG, learning and memory in the STZ-induced SAMP8 mice

**Figure 1:**
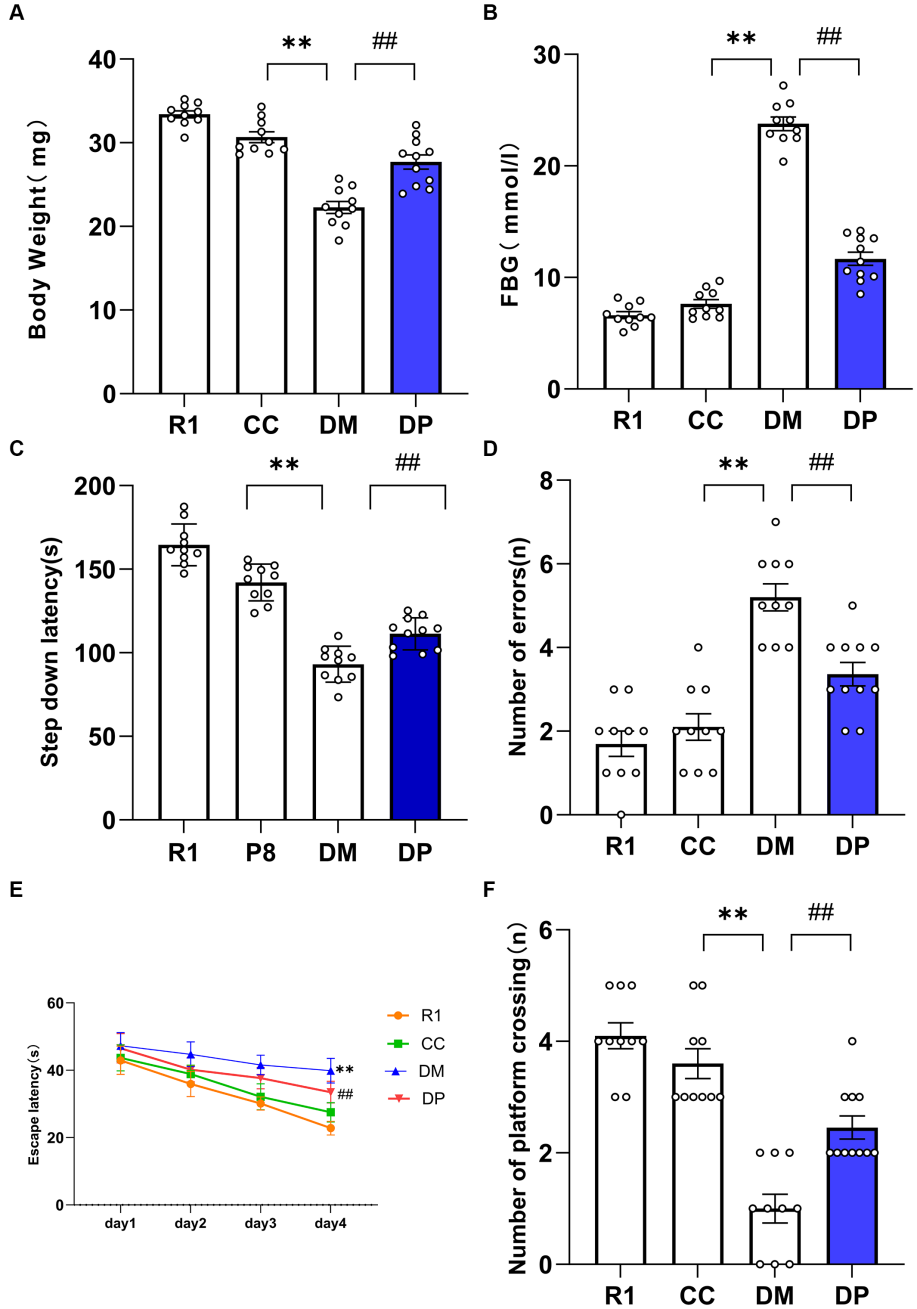
Effects of DP on BW, FBG, and cognitive function in the STZ-induced SAMP8 mice. **(A)** Body weight changes of the mice. **(B)** FBG changes of the mice. **(C)** Latent period changes of the mice. **(D)** The number of errors changes of the mice. **(E)** Escape latency changes of the mice. **(F)** Number of platform crossings after the platform removed changes of the mice. *p < 0.05, **p < 0.01 vs. CC group; ^#^*p* < 0.05, ^##^*p* < 0.01 vs. DM group. R1, control SAMR1 group; CC, control SAMP8 group; DM, STZ induced SAMP8 group; DP, D-pinitol treated STZ induced SAMP8 group. DP, D-pinitol; BW, body weight; FBG, fasting blood glucose. This is a figure. Schemes follow the same formatting.

After 8 weeks of diabetic model establishment, the number of animals that survived in the R1, CC, DM, and DP groups were 10, 10, 10, and 11, respectively. The DM group had a considerably lower BW and higher FBG levels than the CC group (*p* < 0.01). DP treatment resulted in a significant increase in body weight and decrease in FBG levels in STZ-induced SAMP8 mice (*p* < 0 01, Figures 1A,B).

Changes in learning and memory abilities were examined using the step-down and MWM tests. In the step-down test, compared with CC group mice, DM mice had a shorter latent period and a greater number of errors than CC mice (*p* < 0.01). Compared with the DM group, the number of errors decreased and the latent period increased in the DP group (*p* < 0.01; Figures 1C,D). These results suggest that DP can improve learning and memory impairments in DM mice.

The positioning voyage period of MWM is shown in Figure 1E. The latent period of platform search in the DM group was longer than that in the CC group (*p* < 0.01). On the 4th day of training, the escape latency of the DP group was shorter than that of the DM group (*p* < 0.01). During the space exploration period, the number of times the platform was crossed in the DM group was significantly lower than that in the CC group (Figure 1F). The number of mice crossing the platform in the DP group was higher than that in the DM group (*p* < 0.01).

### 3.2. Effects of DP on histological findings of hippocampus in the STZ-induced SAMP8 mice

**Figure 2:**
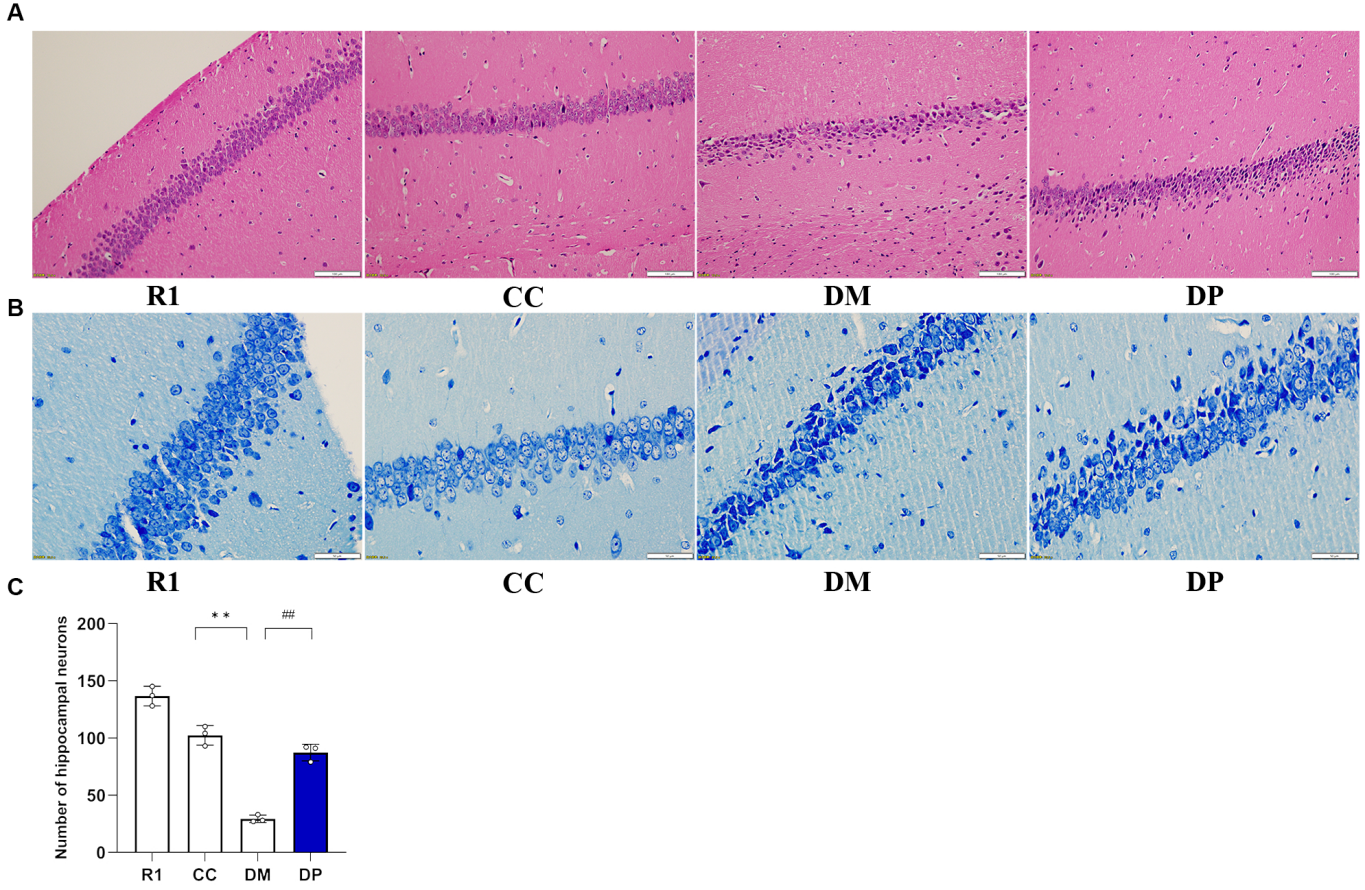
Effects of DP on histological findings of hippocampus in the STZ-induced SAMP8 mice. **(A)** Representative light micrographs of the hippocampus (HE staining, bar: 100 μm). **(B)** Representative light micrographs of the hippocampus (Nissl's staining, bar: 50 μm). **(C)** Quantitative comparison of hippocampal cells in the CA1 region of mice. R1, control SAMR1 group; CC, control SAMP8 group; DM, STZ induced SAMP8 group; DP, D-pinitol treated STZ induced SAMP8 group. **p < 0.01 vs. CC group; ^##^*p* < 0.01 vs. DM group.

As shown in Figure 2A, hippocampal neurons in the R1 group were abundant and had a regular arrangement, and the morphology and structure of the neurons were normal (a round or oval mass). Compared with the R1 group, hippocampal neurons in the CC group were also abundant and arranged regularly, with normal morphology and structure. However, a small amount of cell shrinkage was observed. In the DM group, the neurons were sparse and did not show proper arrangement, with many cells showing shrinkage morphology. The number of surviving neurons in the DP-treated group significantly increased, and they were arranged neatly, accompanied by some shrunken cells. Nissl staining was performed and the samples were observed under a light microscope and quantified. The results showed that the number of CA1 cells in each group was significantly different (Figures 2B,C). Furthermore, neuronal damage was observed in the DM group and the damage caused by DM was partially repaired in the DP group.

### 3.3. Identification of DEPs

**Figure 3:**
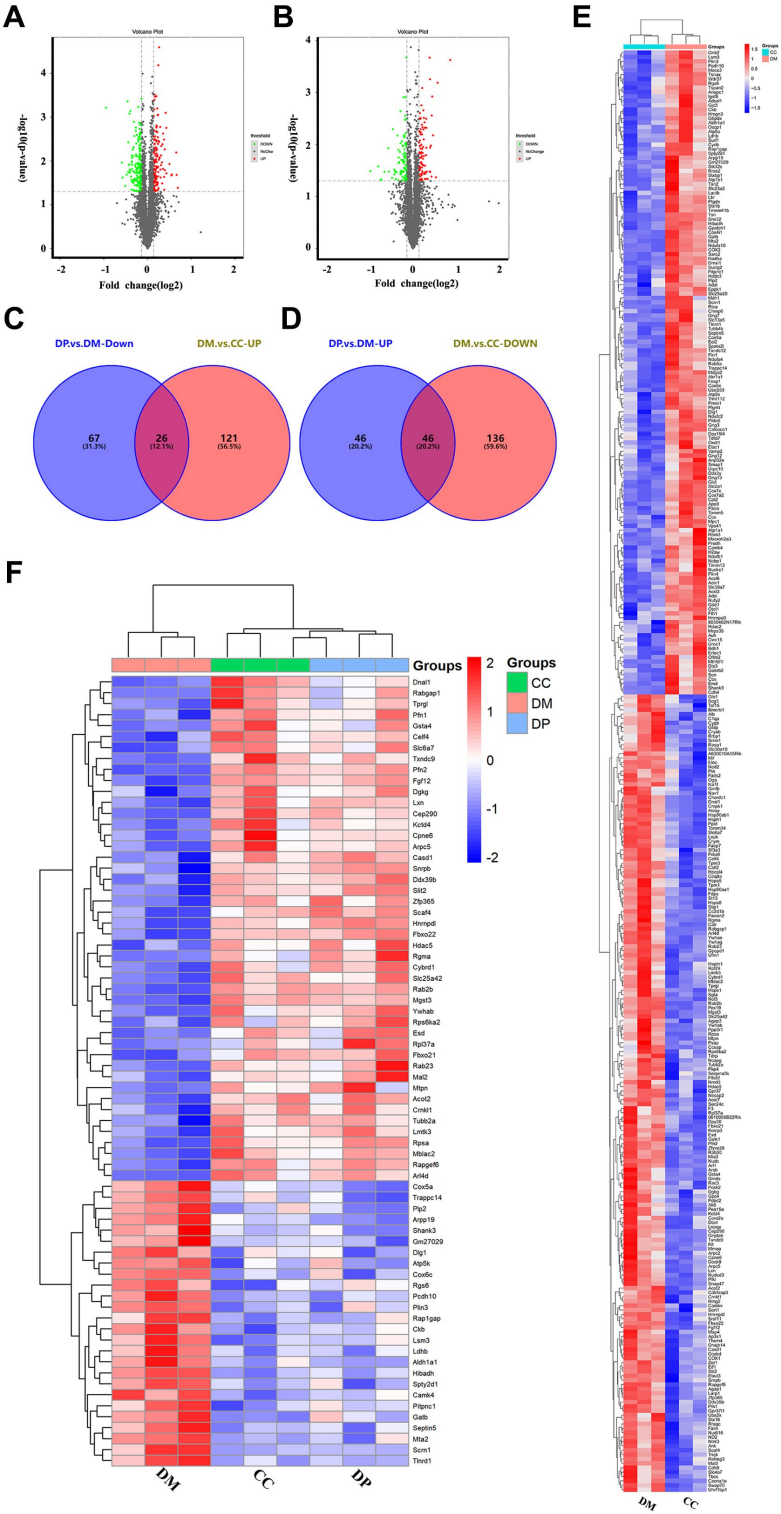
Protein identification and potential targets of DP in treating hippocampal damage. **(A)** volcano plot of DEPs between DM group and CC group. **(B)** Volcano plot of DEPs between DP group and DM group. **(C)** 26 down-regulated proteins after DP therapy in diabetic mice. **(D)** 46 up-regulated proteins after DP therapy in the hippocampus of diabetic mice. **(E)** Cluster heatmap analysis of DEPs between DM group and CC group. **(F)** Cluster heatmap analysis of 72 back-regulated proteins after DP therapy. CC, control SAMP8 group; DM, STZ induced SAMP8 group; DP, D-pinitol treated STZ induced SAMP8 group. DEPs, differentially expressed proteins.

A total of 32,576 unique peptides and 4,570 quantified proteins were identified in the mouse hippocampus (protein FDR ≤ 0.01). These proteins (FC ≤ 0.91 or FC ≥ 1.1, *p* < 0.05) were selected for further screening of DEPs. In this screening, 329 DEPs were identified in the DM group compared to the CC group, including 182 downregulated and 147 upregulated proteins (Supplementary Table S1; Figure 3A). In the DP group, 185 DEPs were identified compared to the DM group, including 93 downregulated and 92 upregulated DEPs (Figure 3B). Of these DEPs, 72 proteins were normalized after DP treatment (Supplementary Table S2), including 26 downregulated and 46 upregulated proteins (Figures 3C,D). Heat maps showed protein gene symbols between the DM and CC groups (Figure 3E). Heat maps showed 72 proteins that were back-regulated after DP therapy (Figure 3F).

### 3.4. Gene ontology (GO) classification and cellular component (CC) analysis of the DEPs

**Figure 4:**
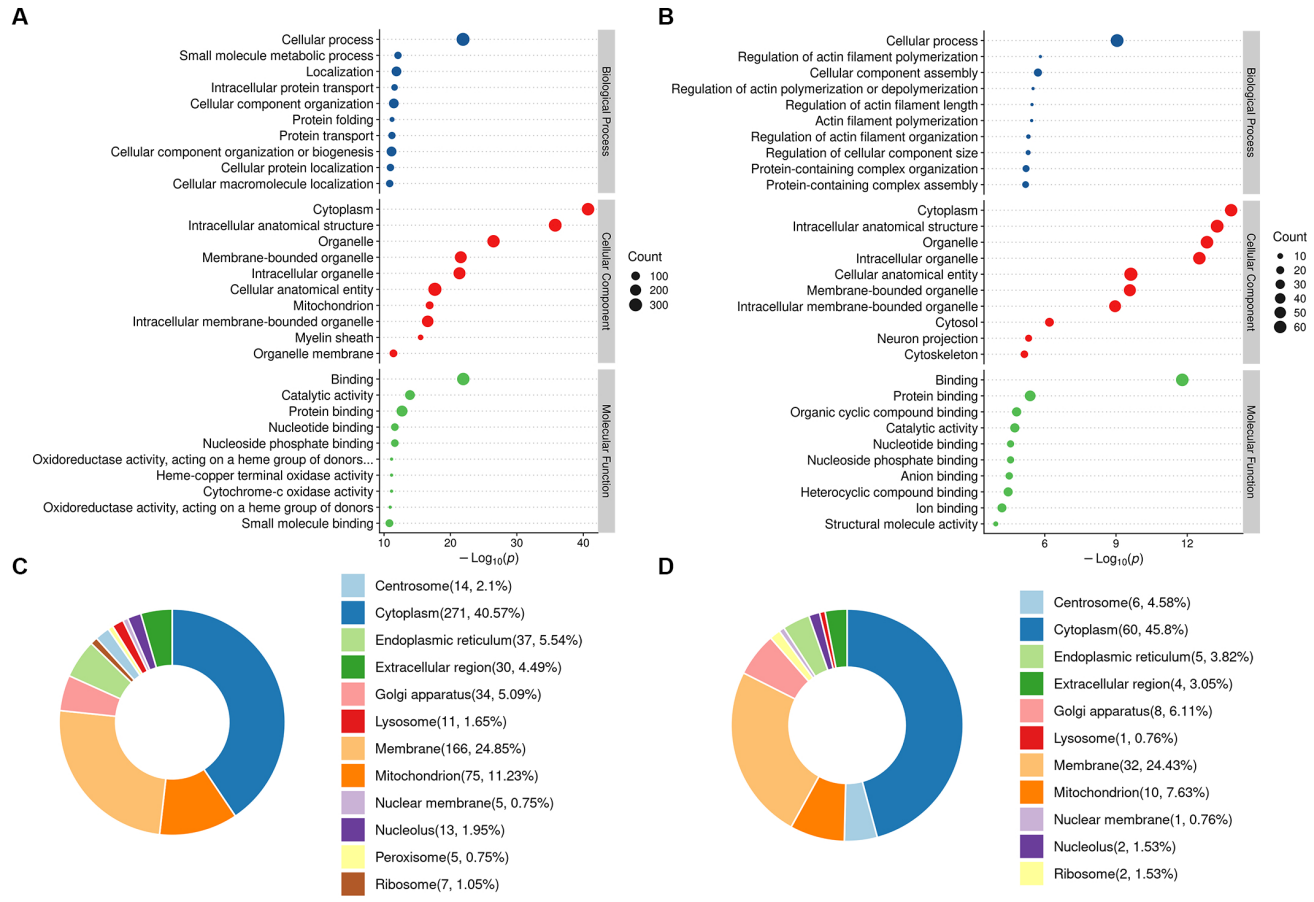
GO function enrichment analysis. **(A)** Identified DEPs between DM group and CC group. **(B)** Identified DEPs between DP group and DM group. **(C)** Subcellular localization of DEPs between DM group and CC group. **(D)** Subcellular localization of DEPs between DP group and DM group. CC, control SAMP8 group; DM, STZ induced SAMP8 group; DP, D-pinitol treated STZ induced SAMP8 group. DEPs, differentially expressed proteins.

GO analysis was performed on the identified DEPs. The top ten generally changed GO terms were expressed using an enrichment bubble map. DEPs in the DM/CC group showed that the cytoplasm was the top of CCs. The main BPs of the enriched DEPs were cellular processes, small molecule metabolic processes, localization, intracellular protein transport, and cellular component organization. According to MF analysis, the DEPs were primarily enriched in binding, catalytic activity, and oxidoreductase activity (Figure 4A). Moreover, proteins normalized to DP treatment were mainly located in the cytoplasm. In the BP analysis, DEPs were mainly enriched in the regulation of actin filament polymerization, cellular protein-containing complex assembly, and actin filament polymerization. The main MF of the enriched DEPs was associated with binding, cytochrome c oxidase activity, and heme-copper terminal oxidase activity (Figure 4B). Subcellular localization analysis showed that the DEPs in the DM/CC group were distributed in the cytoplasm (40.57%), membrane (24.85%), and mitochondrion (11.23%; Figure 4C). DEPs in the DP/DM group were distributed in the cytoplasm (46.1%), membrane (23.4%), and mitochondria (9.22%; Figure 4D).

### 3.5. Multivariate statistical analysis of hippocampus metabolomics of mice

**Figure 5:**
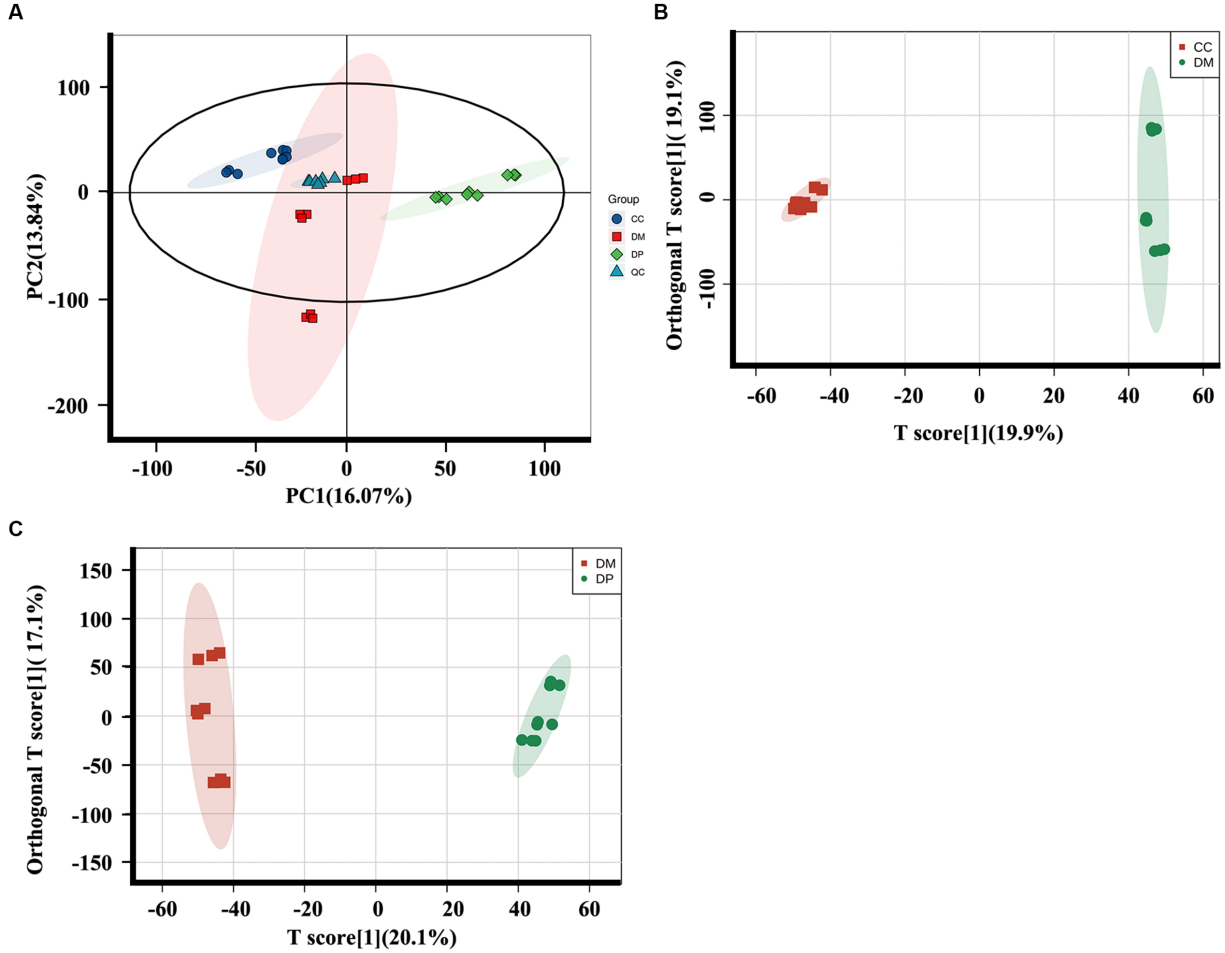
Multivariate statistical analysis of hippocampus metabolomics of mice. **(A)** PCA plot for the CC, DM and DP Groups and QC samples. **(B)** The OPLS-DA plot for the DM group and CC group. **(C)** The OPLS-DA plot for the DP group and DM group (*n* = 9). PCA, principal component analysis; OPLS-DA, orthogonal partial least-squares discriminant analysis; QC, quality control. CC, control SAMP8 group; DM, STZ induced SAMP8 group; DP, D-pinitol treated STZ induced SAMP8 group.

The differential metabolites in each group were analyzed using principal component analysis (PCA) and orthogonal partial least square discriminant analysis (OPLS-DA). PCA performed on all samples revealed that the QC samples were tightly cluster together in the PCA score plots (Figure 5A), indicating excellent QC repeatability and a stable experimental system. The OPLS-DA score plots showed a noticeable difference between the DM and CC groups (R2Y:0.998, Q2:0.94, Q2 Intercept: −1.280; Figure 5B) and the DM and DP groups (R2Y:0.996, Q2:0.951, Q2 Intercept: −1.507; Figure 5C).

### 3.6. Identification of DRMs and HMDB classification

**Figure 6:**
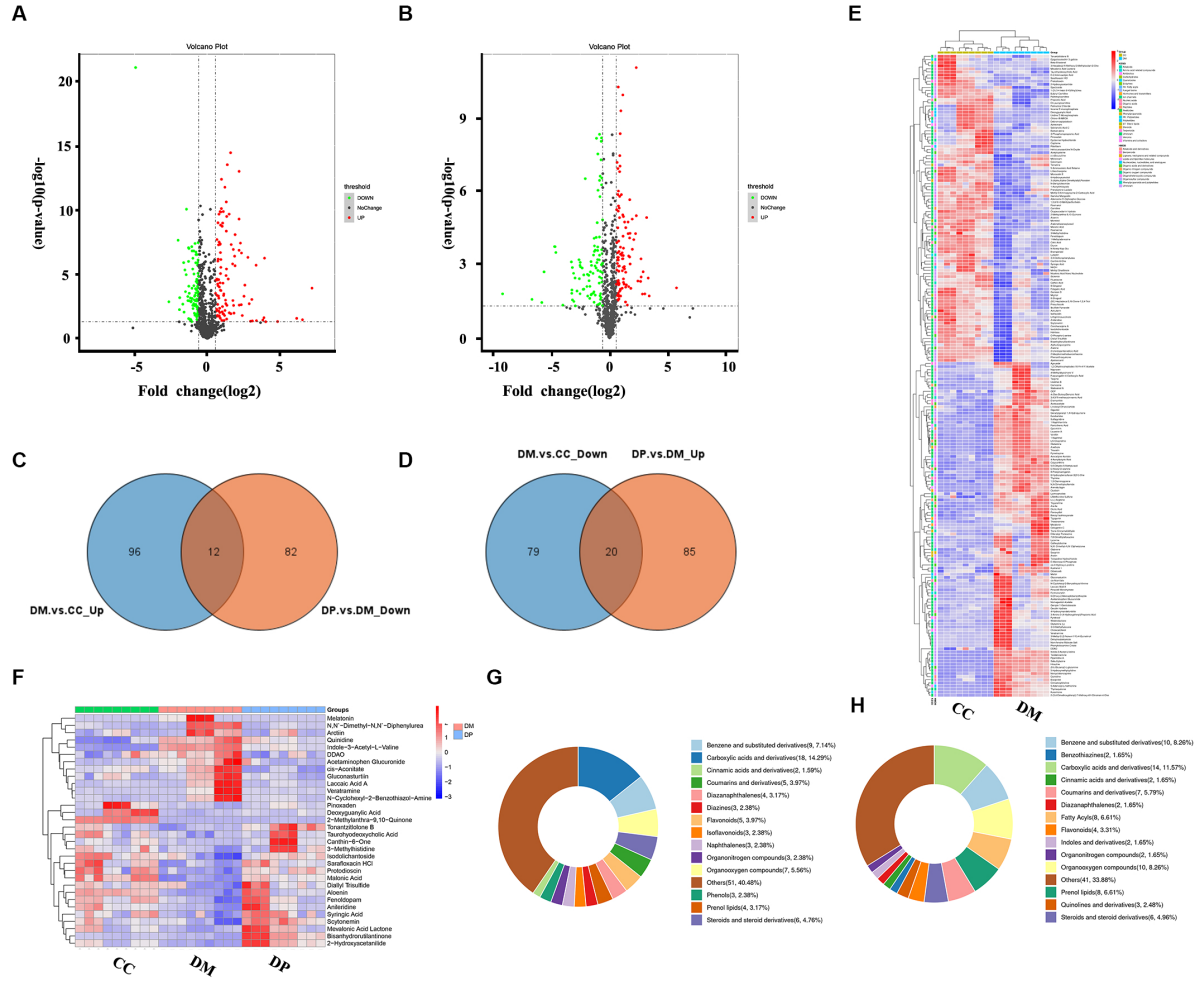
DRMs identification and potential targets of DP in treating hippocampal damage. **(A)** Volcano Plot of DRMs between DM group and CC group. **(B)** Volcano Plot of DRMs between DP group and DM group. **(C)** 20 up-regulated metabolites after DP therapy in the hippocampus of diabetic mice. **(D)** 12 down-regulated metabolites after DP therapy in diabetic mice. **(E)** Cluster heatmap analysis of DRMs between DM group and CC group. **(F)** Cluster heatmap analysis of 32 back-regulated metabolites after DP therapy. **(G)** HMDB classification of DRMs between DM group and CC group. **(H)** HMDB classification of DRMs between DP group and DM group. DRMs, differentially regulated metabolites. HMDB, human metabolome database. CC, control SAMP8 group; DM, STZ induced SAMP8 group; DP, D-pinitol treated STZ induced SAMP8 group.

Metabolic profiling was performed using UPLC-Q-Exactive MS (LC–MS/MS). In total, 832 metabolites were identified. Using a variable importance in projection (VIP) score > 1, *p* < 0.05, and FC > 1.5 or < 0.67, we identified 207 differentially regulated metabolites (DRMs) (108 up-regulated and 99 downregulated) in the DM and CC groups (Figure 6A; Supplementary Table S3), and 199 DRMs (105 up-regulated and 94 downregulated) in the DM and DP groups (Figure 6B). Of these metabolites, 32 DRMs (12 downregulated and 20 upregulated) normalized after DP treatment (Figures 6C,D; Supplementary Table S4). These metabolites include melatonin, veratramine, acetaminophen glucuronide, quinidine, arctiin, deoxyguanylic acid, canthin-6-one, malonic acid, 2-hydroxyacetanilide, syringic acid, protodioscin, fenoldopam, and diallyl trisulfide. Cluster heatmap analysis of 207 DRMs between the DM and CC groups (Figure 6E). Cluster heatmap analysis of the 32 back-regulated metabolites after DP therapy (Figure 6F). According to the Human Metabolome Database (HMDB) classification, carboxylic acids and their derivatives were dominant in the DM and DP groups, accounting for 14.29 and 15.79% (Figures 6G,H).

### 3.7. KEGG pathway enrichment analysis for the DEPs and DRMs

**Figure 7:**
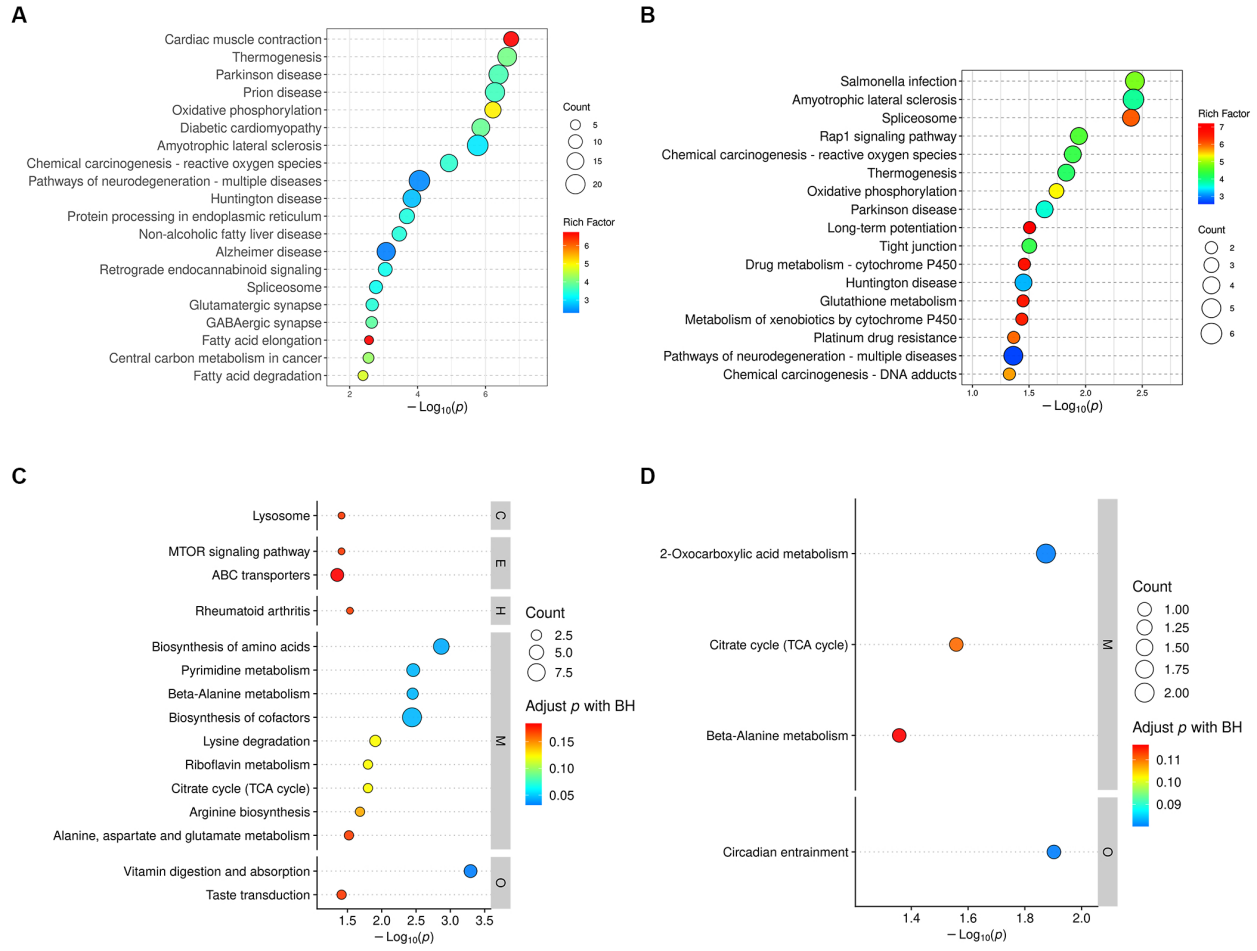
KEGG pathway enrichment analysis. **(A)** KEGG pathways analysis of DEPs between DM group and CC group. **(B)** KEGG pathways analysis of DEPs between DP group and DM group. **(C)** KEGG pathways analysis of DRMs between DM group and CC group. **(D)** KEGG pathways analysis of DRMs between DP group and DM group. The color of the circle indicates the rich factor, and the size of the circle indicates the count. DEPs, differentially expressed proteins. DRMs, differentially regulated metabolites. CC, control SAMP8 group; DM, STZ induced SAMP8 group; DP, D-pinitol treated STZ induced SAMP8 group.

Based on 329 DEPs in the DM group and 72 DEPs that were normalized by treatment with DP, KEGG pathway analysis identified 45 significantly different pathways in the DM group (Supplementary Table S5) and 17 significantly different pathways in the DP group (Supplementary Table S6). The results of the KEGG enrichment analysis demonstrated that the DEPs of the DM group were mainly involved in Alzheimer’s disease, pathways of neurodegenerative diseases, GABAergic synapses, the PPAR signaling pathway, the Ras signaling pathway, oxidative phosphorylation, fatty acid degradation, protein processing in the endoplasmic reticulum, valine, leucine, and isoleucine degradation, pyruvate metabolism, biosynthesis of unsaturated fatty acids, necroptosis, and ferroptosis. The TOP20 pathway is shown in Figure 7A. In addition, the pathways enriched in the DP group mainly included the spliceosome, Rap1 signaling pathway, oxidative phosphorylation, long-term potentiation, tight junctions, glutathione metabolism, and neurodegenerative disease pathways. These pathways are shown in Figure 7B.

KEGG analysis of 207 DRMs in the DM-treated group showed that 15 pathways with significant differences were enriched (*p* < 0.05; Supplementary Table S7). These pathways included vitamin digestion and absorption; riboflavin metabolism; lysine degradation; the citrate cycle (TCA cycle); the mTOR signaling pathway; and alanine, aspartate, and glutamate metabolism (Figure 7C). KEGG analysis was performed on 32 DRMs that were normalized by treatment with DP, and four pathways with significant differences were obtained (*p* < 0.05; Supplementary Table S8), including circadian entrainment, citrate cycle (TCA cycle), and beta-alanine metabolism (Figure 7D).

### 3.8. Protein–protein interaction (PPI) network analysis

**Figure 8:**
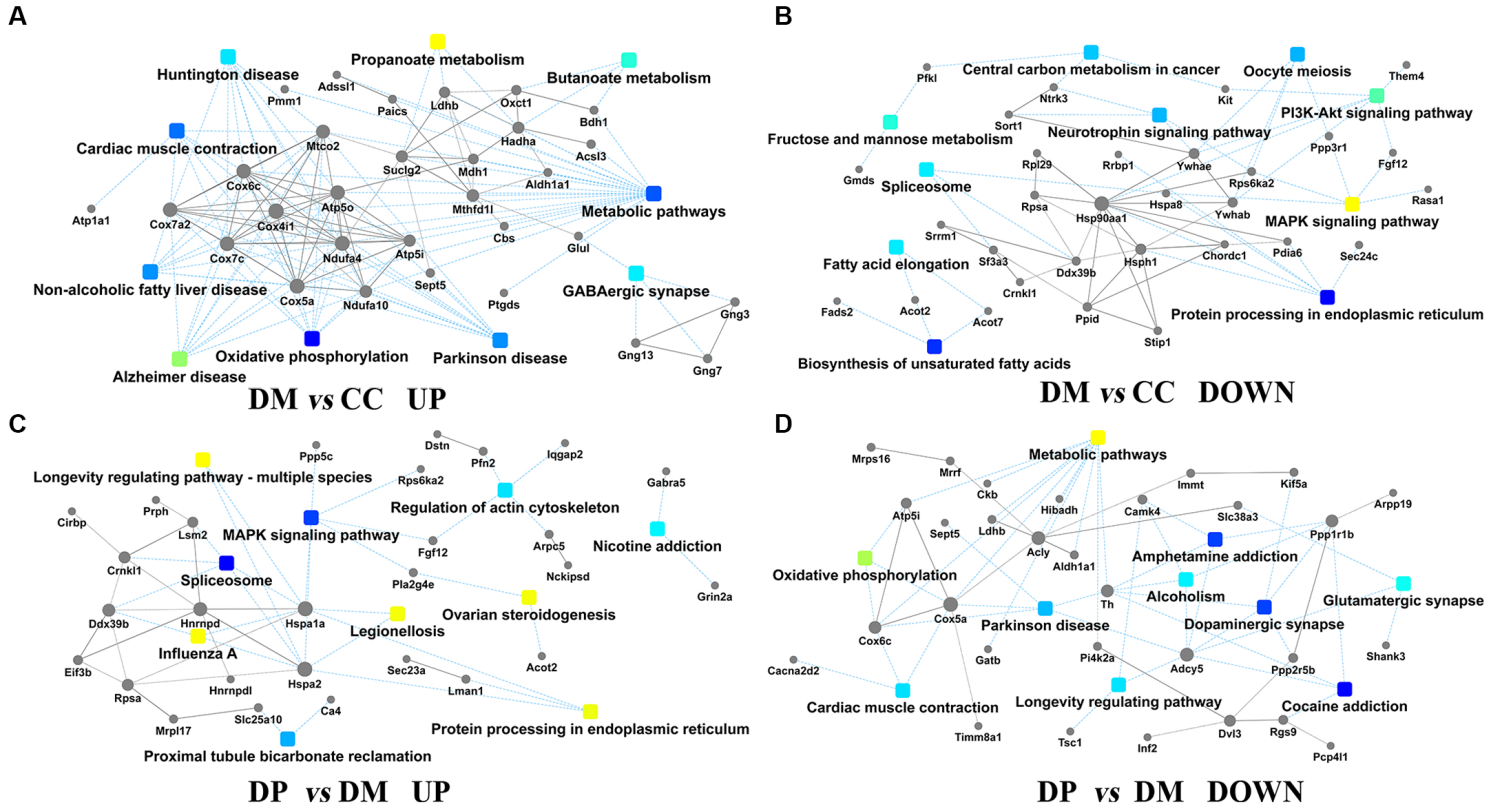
PPI network analysis. **(A)** PPI pathway-gene network between DM group and CC group (up-regulated). **(B)** PPI pathway-gene network between DM group and CC group (down-regulated). **(C)** PPI pathway-gene network between DP group and DM group (down-regulated). **(D)** PPI pathway-gene network between DP group and DM group (up-regulated). DEPs, differentially expressed proteins; PPI, protein-protein interaction. CC, control SAMP8 group; DM, STZ induced SAMP8 group; DP, D-pinitol treated STZ induced SAMP8 group.

PPI analysis revealed the protein interaction network of the DEPs in the CC, DM, and DP groups (Figures 8A–D). Hsp90aa1, Cox4i1, Acly, and Hspa1a were located at the core of the PPI network. These subnetworks were related to metabolic pathways (*n* = 28), oxidative phosphorylation (*n* = 10), Alzheimer’s disease (*n* = 9), protein processing in the endoplasmic reticulum (*n* = 6), PI3K-Akt signaling pathway (*n* = 6), MAPK signaling pathway (*n* = 5), and GABAergic synapses (*n* = 5) in the DM group. However, in the DP group, these subnetworks were related to metabolic pathways (*n* = 7), the spliceosome (*n* = 4), the Rap1 signaling pathway (*n* = 4), regulation of the actin cytoskeleton (*n* = 3), and oxidative phosphorylation (*n* = 3).

### 3.9. Western blot validation of selected DEPs

**Figure 9:**
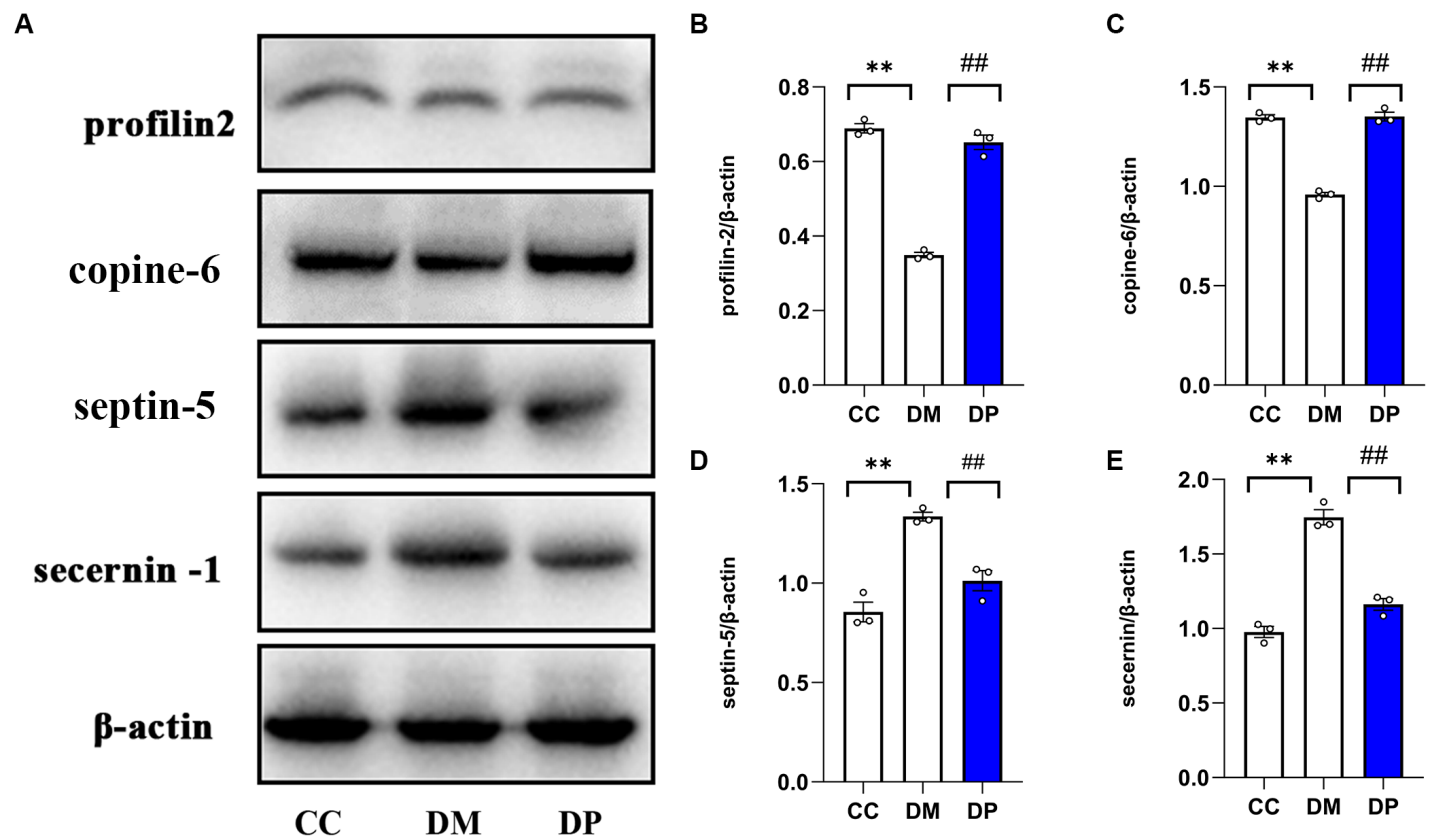
Western blot validation of selected DEPs. **(A)** Representative western blot images of profilin2, Copine-6, Septin5, and secernin4. **(B–E)** Data were expressed as the expression ratio of profilin2/β‐actin, Copine-6/β‐actin, Septin5/β‐actin, and secernin4/β‐actin. *p < 0.05, **p < 0.01 vs. CC group; ^#^*p* < 0.05, ^##^*p* < 0.01 vs. DM group. DEPs, differentially expressed proteins. CC, control SAMP8 group; DM, STZ induced SAMP8 group; DP, D-pinitol treated STZ induced SAMP8 group.

Profilin 2, copine-6, septin-5, and secernin-4 were selected for WB validation studies based on the significance of the differences, relationship with the nervous system, and availability of relevant antibodies. Western blot analysis (Figure 9A) showed that, compared with the DM group, septin-5 and secernin 4 levels in the DP group were downregulated, while profilin 2 and Copine-6 levels were upregulated, and the difference was significant (Figures 9B–E). The validation results are consistent with the TMT analysis results.

### 3.10. Integration analysis of proteome and metabolic data

**Figure 10:**
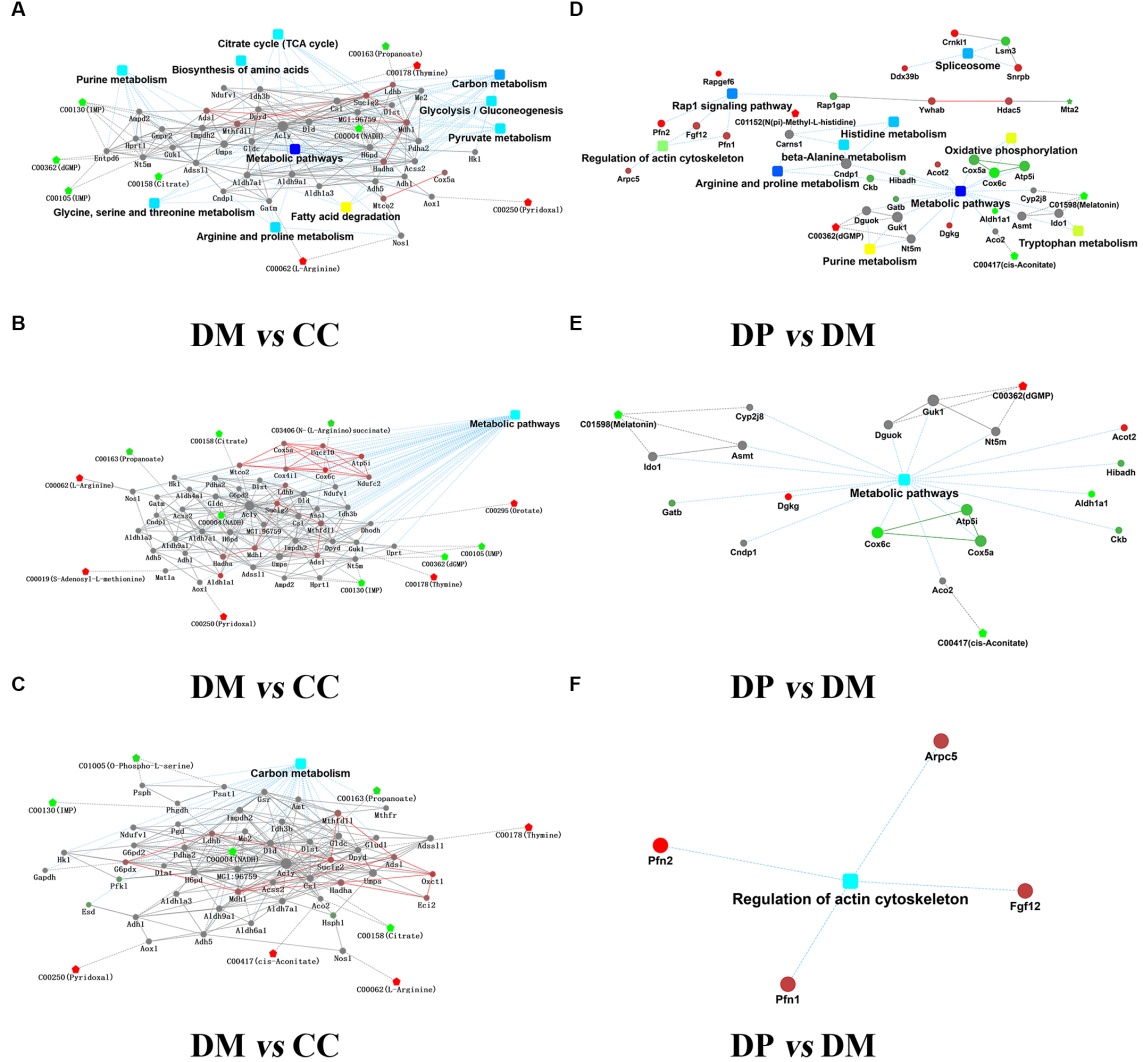
Integration analysis of proteome and metabolic. **(A)** Overview of PPI network of DEPs-DRMs between DM group and CC group. **(B)** PPI for DEPs-DRMs of metabolic pathways between DM group and CC group. **(C)** PPI for DEPs-DRMs of Carbon metabolism pathways between DM group and CC group. **(D)** Overview of PPI network of DEPs-DRMs between DP group and DM group. **(E)** PPI for DEPs-DRMs of metabolic pathways between DP group and DM group. **(F)** PPI for DEPs-DRMs of Regulation of actin cytoskeleton between DP group and DM group. Rectangular nodes indicate KEGG Pathway, Circular nodes represent proteins/genes, and pentagons represent metabolites (up-regulation in red and down-regulation in green). DEPs, differentially expressed proteins. DRMs, differentially regulated metabolites. CC, control SAMP8 group; DM, STZ induced SAMP8 group; DP, D-pinitol treated STZ induced SAMP8 group.

R 4.0.1 and Cytoscape 3.8.2 were used for network analysis of DEPs and DRMs. Based on 329 DEPs and 207 DRMs in the DM group, Correlation analysis of DEPs-DRMs revealed that nicotinamide adenine dinucleotide (NADH) was significantly correlated with the expression of 78 proteins between the DM and CC groups (VIP = 1.503, FC = 0.39998, *p* < 0.001). Network analysis showed that the pathways mainly included carbon metabolism (TCA cycle, glycolysis/gluconeogenesis, pyruvate metabolism), purine metabolism, biosynthesis of amino acids (glycine, serine, and threonine metabolism, arginine and proline metabolism, and fatty acid degradation; Figures 10A–C).

Based on 78 DEPs and 32 DRMs that were normalized by treatment with DP, correlation analysis of DEPs-DRMs showed that melatonin was significantly correlated with the expression of 3 proteins between DP group and DM group (VIP = 1.219, FC = 0.010227, *p* < 0.05). Network analysis showed that the pathways mainly included metabolic pathways (purine metabolism, arginine and proline metabolism, histidine metabolism), regulation of the actin cytoskeleton, oxidative phosphorylation, Rap1 signaling pathway, and others (Figures 10D–F).

## 4. Discussion

DCI is a complication of diabetes that has attracted considerable attention in recent years. This neurodegenerative disease is mainly manifested by cognitive dysfunction, which not only seriously affects the blood glucose control level and quality of life in patients but also increases the risk of Alzheimer’s disease. The hippocampus is an important anatomical structure involved in learning, memory, and other cognitive functions. Therefore, the hippocampus is a key brain region in the study of diabetic cognitive dysfunction. Previous studies have shown that hyperglycemia can lead to hippocampal atrophy and neuronal apoptosis (Sadeghi et al., 2016; Li et al., 2020; Gupta et al., 2023). Now, pathogenesis and treatment of diabetes-induced hippocampal damage and DCI has become a hotspot of modern medical. In this study, we created a diabetic aging mouse model by administering STZ intraperitoneally to SAMP8 mice for five consecutive days. Using a light microscope, we found a significant reduction in the number of hippocampal neurons and an abnormal morphology of these neurons in the hippocampus of SAMP8 diabetic mice, indicating hippocampal damage. The Morris water maze and step-down tests were used to evaluate cognitive function and cognitive impairment was observed in the diabetic mice. We also observed that administration of DP significantly reduced blood glucose levels in diabetic mice and ameliorated diabetes-induced hippocampal neuronal damage, suggesting that DP has a protective effect on the hippocampus in diabetes. To explore the precise pathogenesis of diabetes-induced hippocampal damage and how DP effectively mitigates this damage, we conducted research using proteomic and metabolomic approaches.

Firstly, we performed proteomic analysis of the hippocampus of SAMP8 mice with STZ-induced diabetes. Among the 72 proteins normalized by DP treatment, septin5 was up-regulated in the hippocampal tissue of the DM group and downregulated by DP administration. Septin is a member of the GTPase superfamily and binds GTP (DeMay et al., 2011). Neurodegenerative disorders, such as Alzheimer’s and Parkinson’s diseases, have been linked to abnormal septin function (Peterson and Petty, 2010). One, a member of the septin family, Septin5, is speculated to promote neurofibrillary tangle development based on its function in tau-based paired helical filament cores (Son et al., 2005). Downregulation of septin5 enhances autophagosomal activity and reduces amyloid β (Aβ) peptide levels in neuronal cells (Marttinen et al., 2020). Autophagy inhibition can further aggravate cognitive impairment, whereas enhanced autophagy alleviates symptoms in animal models of diabetic encephalopathy (Cheng et al., 2022). Thus, DP enhances neuronal autophagy and regulates Aβ peptide production by decreasing septin5 levels to ameliorate diabetes-induced hippocampal damage. Secernin-1 (SCRN1) is a cytosolic protein that regulates exocytosis in rat peritoneal mast cells (Way et al., 2002). Transcriptomic and proteomic studies have shown that the mRNA and protein expression levels of SCRN1 in the brain are higher than those in other tissues (Fagerberg et al., 2014; Uhlén et al., 2015). The proteomics results revealed that SCRN1 expression was detected in the hippocampus of the AD group but not in the normal control group (Ayyadevara et al., 2016). In studies of diseases related to cognitive impairment such as AD and primary age-related tauopathy, many SCRN1 aggregates were found in NFTs and plaque-related dystrophic neuritis (Pires et al., 2019). One study identified SCRN1 as an early marker of neurodegeneration in transgenic mice overexpressing human tau protein (Chang et al., 2013). In our study, SCRN1 was significantly upregulated in the DM group and was downregulated after DP treatment.

Additionally, CPNE6 was downregulated in the hippocampal tissue from the DM group and upregulated by DP treatment. Copines (CPNEs) belongs to a group of cytoplasmic proteins that bind phosphatidylserine at micromolar levels of calcium and promote lipid vesicle aggregation (Creutz et al., 1998). The CPNEs family consists of nine members. Among these, CPNE6, also known as the “N-copine,” is specifically expressed in brain tissue (Perestenko et al., 2010). *In situ* hybridization analysis revealed that N-copine mRNA is only expressed in hippocampal neurons and in the main and accessory olfactory bulbs, which are the main sites of synaptic plasticity and memory formation (Nakayama et al., 1999). Presumably, memory is encoded by modification of synaptic strength through cellular mechanisms such as long-term potentiation (LTP) and long-term depression (LTD) (Bin Ibrahim et al., 2022). In the Schaffer collateral pathway of hippocampal slices, depletion of postsynaptic CPNE6 results in decreased LTP and increased LTD (Burk et al., 2018). It has been demonstrated that CPNE6 is related to the remodeling of dendritic spines (Reinhard et al., 2016). When CPNE6 is overexpressed, mushroom spines increase, and filopodia decrease in hippocampal neurons (Burk et al., 2018). Spine density and mushroom spines are associated with spatial and working memory (Mahmmoud et al., 2015). It may be that DP promotes the remodeling of dendritic spines. The actin-binding protein profilin (PFN) was isolated from calf spleen in 1997 (Carlsson et al., 1977). Since then, four PFN genes (PFN1-4) in mammalian genomes have been identified in the mammalian genome. Among these, PFN2 is expressed mainly in the brain (Di Nardo et al., 2000). The cytoskeletal protein profilin 2 and its upstream effector RhoA/ROCK are involved in the assembly of actin rods in the SMA. Actin rod formation has harmful effects on motor neuron homeostasis by affecting actin dynamics and interfering with essential cellular pathways (Walter et al., 2021). However, other studies have shown that PFN2 is not required for neurite extension or neuronal polarization (Pilo Boyl et al., 2007). In the present study, PFN2 was downregulated in the DM group and upregulated after the DP intervention. Further research is necessary to elucidate the role and mechanism of PFN2 in the nervous system.

Secondly, we conducted LC–MS-based metabolomics to investigate molecular changes in the hippocampi of DM mice and DP-treated mice. Among the 32 DRMs normalized after DP treatment, we selected DCI-related metabolites for discussion based on literature reports. Veratramine is extracted from the natural Lilium veratrum family (Khanfar and El Sayed, 2013), and *Veratrum* alkaloids can cause DNA damage in the cerebellum and cerebral cortex of mice via oxidative stress (Cong et al., 2007). In our study, veratramine was upregulated in the hippocampi of the DM group and downregulated by DP treatment. Melatonin is a critical circadian hormone secreted by the pineal gland and has been widely studied for its complex role in diabetes. It was reported that melatonin administration for 3 or 6 weeks reduced serum insulin levels and homeostasis model assessment (HOMA) index in obese rats (Nduhirabandi et al., 2014). Other experimental studies have reported that plasma melatonin levels increase in STZ-induced diabetic rats and are associated with decreased insulin levels (Peschke et al., 2008). One study enrolled 30 patients with type 2 diabetes and gave them melatonin therapy for 7 days. The results showed that melatonin supplementation increased glycemic variability in patients with T2DM (Martorina and Tavares, 2023). However, some studies have suggested that melatonin is effective in treating diabetes. A simultaneous *in vivo* and *in vitro* study has shown that combination treatment with melatonin and sitagliptin is superior to monotherapies in treating type 2 diabetes (T2D) (Patel et al., 2023). A recent study showed that melatonin-loaded chitosan/lecithin (Mel-C/L) demonstrated multiple effects in diabetic rats, including reducing blood glucose levels, promoting beta cell regeneration, and having anti-inflammatory, anticoagulant and antioxidant properties (Alaa et al., 2023). In our study, melatonin was significantly upregulated in the DM group and was downregulated after DP treatment. Based on the above studies, it is speculated that there may be a U-shaped relationship rather than a linear relationship between melatonin and diabetes.

In this study, syringic acid (SA) was downregulated in the DM group and upregulated by DP treatment. SA is a natural phenolic compound derived from plants (Arumugam et al., 2018). Several studies have shown that SA protects the brain, hippocampus, and other brain regions through its antioxidant and anti-inflammatory properties (Güzelad et al., 2021; Mirza et al., 2022). Butyric acid can improve hippocampal deficits and cognitive functions such as learning and memory (Ogut et al., 2022). Increasing the concentration of SA may be one of the mechanisms by which DP ameliorates diabetic hippocampal injury and cognitive impairment. Diallyl trisulfide (DATS) is an organic sulfide compound extracted from garlic and known for its potent antioxidant properties (Liu et al., 2006). DATS can reverse or reduce DOX-induced neurotoxicity in the cerebral cortex and hippocampus, possibly related to its anti-inflammatory and antioxidant effects (Leung et al., 2020). A previous study demonstrated that DATS mediates cell survival by enhancing autophagy and activating the Nrf2/antioxidant response element pathway (Liu et al., 2018). The organosulfur compounds were detected in black garlic by GC–MS, including DATS (203.9 μg/g), which can improve rats’ learning and memory ability by decreasing the activity of AChE (Chen et al., 2021). In our study, DATS was downregulated in the DM group and upregulated by DP treatment.

Finally, in order to further explore the pathogenesis of diabetes-induced hippocampal injury and the mechanism of DP ameliorated diabetes-induced hippocampal injury, we applied TMT-based proteomics and LC–MS-based metabolomics to comprehensively analyze hippocampal damage induced by diabetes and the protective effect of DP on the diabetic hippocampus. Correlation analysis of DEPs-DRMs showed that NADH may be an essential metabolite in the pathogenesis of DCI. NADH is one of the primary molecules of energy transfer in the mitochondrial respiratory chain and is a vital parameter of cellular energy metabolism. The research on NADH and central nervous system diseases is increasing. One study reported that injection of neurotoxic 5, 7-dihydroxytryptamine into the dorsal raphe nucleus in the brainstem resulted in a 50% decrease in NADH fluorescence in the injection area, indicating no recovery of metabolic activity (Rex et al., 2001). It has been shown that aging reduced the mitochondrial free NADH concentration by 43% in NTg neurons and 50% in 3xtg-AD (Dong et al., 2019). It has been demonstrated that early oxidative damage leads to decreased NADH and ATP levels (Galino et al., 2011). Research employing label-free resonance Raman spectroscopy (RRS) has shown a significant correlation between mitochondrial REDOX imbalance, specifically the mitochondrial NAD+/NADH ratio, in cultured neurons and organotypic cortical slices exposed to high glucose levels. This NAD+/NADH ratio may be an early predictor of cognitive impairment in diabetes (Wu et al., 2022). In our study, NADH was lower in the DM group than in the CC group. Meanwhile, we found that energy metabolism imbalance was the main cause of diabetes-induced hippocampal damage, including carbon metabolism (TCA cycle, glycolysis/gluconeogenesis, pyruvate metabolism), purine metabolism, biosynthesis of amino acids (glycine, serine, and threonine metabolism, arginine and proline metabolism, fatty acid degradation, necroptosis, and ferroptosis).

Correlation analysis of DEPs-DRMs showed that melatonin may be a critical target for DP to alleviate DCI. Abnormal melatonin secretion affects blood glucose levels (Martorina and Tavares, 2023) and circadian rhythm. Hyperglycemia and circadian rhythm disorder are risk factors for DCI (Barone et al., 2021; Liu et al., 2023). DP can regulate the secretion of melatonin and improve the risk factors of cognitive function, which may be one of the targets of DP in improving diabetic cognitive impairment. Further network analysis found that DP alleviated diabetes-induced hippocampal damage by modulating metabolic pathways (purine metabolism, arginine and proline metabolism, histidine metabolism), actin cytoskeleton regulation, oxidative phosphorylation, long-term potentiation, and the Rap1 signaling pathway.

## 5. Conclusion

In this study, we applied TMT-based proteomics and LC–MS-based metabolomics to comprehensively analyze hippocampal damage induced by diabetes and the protective effect of DP on the diabetic hippocampus. We found that NADH may be an essential metabolite in the pathogenesis of DCI. Energy metabolism imbalance was the leading cause of diabetes-induced hippocampal damage, including carbon metabolism, purine metabolism, biosynthesis of amino acids, fatty acid degradation and cell death (necroptosis and ferroptosis). Melatonin may be one of the targets of DP in improving diabetic cognitive impairment. DP alleviated diabetes-induced hippocampal damage by rebalancing metabolic pathways (purine metabolism, arginine and proline metabolism, histidine metabolism), actin cytoskeleton regulation, oxidative phosphorylation, long-term potentiation, and the Rap1 signaling pathway. Our study contributes to an enhanced understanding of the mechanism behind diabetes-induced hippocampal damage and cognitive impairment and provides a potential target for the protective effects of DP.

Although our research obtained certain results, there are also some deficiencies. First, the total number of included samples was relatively small, which may have affected the interpretation of results. Second, only four differential proteins were verified by WB; the proportion of verified proteins was relatively low, so there may be some omissions.

## Data availability statement

The original contributions presented in the study are included in the article/Supplementary material, further inquiries can be directed to the corresponding author/s.

## Ethics statement

The animal study was approved by the Animal Protection and Use Committee of Shandong University. The study was conducted in accordance with the local legislation and institutional requirements.

## Author contributions

XXL: writing original draft, methodology, obtaining data, bioinformatics analysis of proteomics and metabolomics, and review and editing. YG: writing original draft and methodology. BL: editing-original draft, data analysis, and review and editing. WZ: performed experiments and data analysis. QC, WY, and SZ: data analysis. XLL: review manuscript and design. HG and MC: review manuscript, design, methodology, funding acquisition, and management. All authors read and approved the final manuscript.
