# Supplementary material for: Integrated proteomics and metabolomics analysis of D-pinitol function during hippocampal damage in streptozocin-induced aging-accelerated mice
Source: Front Mol Neurosci. 2023 Oct 30;16:1251513. doi: 10.3389/fnmol.2023.1251513 (PMC10664147; doi:10.3389/fnmol.2023.1251513)
Supplement: Supplementary file 1 [file Presentation_1.zip › Supplementary material.docx]

***Supplementary Material***

# Supplementary Methods

## Animals

Male senescence-accelerated prone 8 (SAMP8, n = 40, 12 weeks old) and senescence-accelerated mouse resistant 1 (SAMR1, n = 10, 12 weeks old) were provided by Peking University Laboratory Animal Centre (Beijing, China). The experimental animals were fed in the standardized feeding room of the experimental animals. The temperature is 20-22℃ and the humidity is 55±5%. Mice are free to drink tap water and eat pellet chow. The mice were adaptively pre-fed for 1 week before the experiment. SAMR1 and SAMP8 mice were randomly divided into SAMR1 control group (CC1, n = 10), SAMP8 control group (CC2, n = 10), which injected intraperitoneally with 0.1mol/L citrate buffer. The other SAMP8 mice were received with freshly prepared 0.1mol/L citrate buffer (pH 4.5) to dissolve STZ (50 mg/kg) for 5 consecutive days. Only the mice with fasting blood glucose ≥ 16.7 mmol/L after 1 week were considered to be successful diabetic models and the follow-up experiments were carried out. The blood glucose of 4 mice was lower than 16.7 mmol/L, and the study was excluded. After successful establishment of the model, diabetic mice were divided into DM group (n = 13) and DP group (n = 13). The mice in DM group were given the same volume of double distilled water daily for 8 weeks, while those in DP group were given intragastric administration of DP 150mg/kg/day for 8 weeks. After the behavior experiment, the mice were killed under pentobarbital anesthesia. The hippocampal tissues were collected and kept at -80℃.

## Proteomics Analysis

### Sample Preparation

The hippocampus tissue was remelted at 4 ℃ and grinded with liquid nitrogen, and then 400 μL SDT lysate was added to each sample at 95 ℃ 3min, then ultrasonic 2 min at 4 ℃ and 16000g centrifugal 20min. The supernatant was extracted and protein was quantified by BCA.

In each group, 15 μg protein samples were added to 5X buffer at 5:1 (v / v) and 5min was carried out in boiling water for 8-16%SDS-PAGE electrophoresis. Coomassie brilliant blue staining. Take 300 μg of each sample for enzymatic hydrolysis, the steps are as follows: each sample was added DTT to 100mM, boiling water bath 5min, cooling to room temperature. Add 200 μL UA buffer (8m Urea,150mM Tris-HCl, pH8.0) to mix, transfer to 10KD ultrafiltration centrifuge tube, centrifuge 12000g 15min. discard the filtrate and add 200 μL UA buffer to centrifuge 12000g 15min. Add 100 μL IAA (50mM IAA in UA), 600rpm oscillate 1min, avoid light at room temperature 30min, centrifuge 12000g 10min. Add 100 μL UA buffer and centrifuge 12000g 10min twice. Add 100 μL NH4HCO3 buffer and centrifuge 14000g 10min twice. Adding 60 μL Trypsin buffer (6 μg Trypsin in 40 μL NH4HCO3 buffer), 600rpm oscillates for 1 min at 37 ℃ for 16-18 h. Take a new collection tube, centrifuge 12000g 10min, collect the filtrate, add an appropriate amount of 0.1% TFA solution to resolute, use Thermo desalting spin column desalting treatment, and quantify the peptide using microplate reader (Thermo Scientific). TMT peptide labeling and peptide grading.

The same number of peptides were taken from each sample and labeled according to the instructions of Thermo Fisher TMT labeling kit. The labeled peptides in each group were mixed in the same amount, and the dried peptides were separated by Pierce reversed High pH Reversed-Phase Peptide Fractionation Kit, Thermo Fisher. Finally, the sample collection was merged into 10 components. The peptides of each component were dried and resoluted with 0.1% FA for LC-MS analysis.

### LC-MS/MS analysis

An appropriate amount of peptides from each sample was separated by nano-liter flow rate Easy nLC 1200 chromatography system (Thermo Scientific). Buffer: solution A is 0.1% formic acid aqueous solution, and solution B is a mixture of 0.1% formic acid, acetonitrile and water (of which acetonitrile is 95%). The chromatographic column was balanced with 100% liquid A. The sample was injected into Trap Column (100 μ m * 20mm, 5 μ m, C18 Dr Maisch GmbH) and then separated on a chromatographic column (75 μm * 150mm, 3 μm, C18 Dr. Maisch GmbH) with a flow rate of 300 nl/min.The liquid phase separation gradient is as follows: 0-2 min, B liquid linear gradient from 2% to 8%; 2-71 min, B liquid linear gradient from 8% to 28%; 71-79 min, B liquid linear gradient from 28% to 40%; 79-81 min, B liquid linear gradient from 40% to 100%; 81--90 min, the concentration of B liquid is maintained at 100%. The peptides were separated and analyzed by DDA (data dependent acquisition) mass spectrometry with Q-Exactive HF-X mass spectrometer (Thermo Scientific). The analysis time is 90 min, the detection mode is positive ion, the scanning range of parent ion is 400-1800m/z, the resolution of first-order mass spectrometry is 60000 @ m/Z200, target: 3e6, the first-order Maximum IT:50 ms. The secondary mass spectra of peptides were collected according to the following methods: after each full scan (full scan), the secondary mass spectra (MS2 scan) of 20 highest intensity parent ions were triggered and collected. The resolution of secondary mass spectrometry was 45000 @ m/z 200, AGC target: 1e5, secondary Maximum IT:50 ms, MS2 Activation Type: HCD, Isolation window: 1.2m/z, Normalized collision energy32.

### Database retrieval

The original RAW file of LC-MS/MS is imported into the search engine Sequest HT in the Proteome Discoverer software (version 2.4, Thermo Scientific) for database retrieval. The database used in the database is uniprot-Mus musculus (Mouse) [10090]-88079-211124, which is derived from the network address https://www.uniprot.org/taxonomy/10090 protein database, its protein entry: 88079.

## Metabolomics analysis

### Metabolite extraction

Each sample was weighed 60mg, 200 μ L pre-cold water and 800 μ L pre-cooled methanol acetonitrile were added and mixed evenly. The supernatant was obtained by ultrasonic 1h in ice bath and placed at -20 ℃ for 2h and centrifuged at 16000g / 4 ℃. The supernatant is evaporated in a high-speed vacuum concentration centrifuge. During the mass spectrometry detection, 150 μ L methanol solution was added and centrifuged at 20000g4 ℃ for 40min. The supernatant was collected and analyzed.

### QC reparation

The QC sample is a mixture of all samples in the same amount. QC samples are used to evaluate the accuracy and sensitivity of instruments, as well as the consistency and stability of experimental procedures.

LC-MS/MS mass spectrometry

During the whole analysis process, the sample was placed in an automatic injector at 4 ℃, and the sample was separated by SHIMADZU-LC30 ultra high-performance liquid chromatography (UHPLC) and HILIC column. The injection volume is 3 μL, the column temperature is 25 ℃, the flow rate is 0.3 mL / min, the chromatographic mobile phase A: water + 25mM ammonium acetate, B: acetonitrile, the chromatographic gradient elution procedure is as follows:0-1min,95% B;1-7min,B changes linearly from 95% to 65%;7-9min,B changes linearly from 65% to 35%; 9-10.5 min, B holds at 35%;10.5-11min,B changes linearly from 35% to 95%;11-15min,B holds at 95%.

Positive ion (+) and negative ion (-) patterns were detected by electrospray ionization (ESI) in each sample. After UPLC separation, the samples were analyzed by QEPlus mass spectrometer (Thermo Scientific) and ionized by HESI source. The ionization conditions are as follows: SprayVoltage:3.8kv (+) and 3.2kv (-). Capillary Temperature:320(±); Sheath Gas:30(±); Aux Gas: 5(±); Probe Heater Temp:350(±); S-Lens RF Level:50. The mass spectrometry acquisition setting is as follows: mass spectrometry acquisition time: 12min. The scanning range of parent ion is 80-1200m/z, the resolution of primary mass spectrometry is 70000@m/z 200, AGC target:3e6, primary Maximum IT:100 ms. The second-order mass spectrometry was collected according to the following methods: after each full scan, the second-order mass spectrometry (MS2 scan) was triggered to collect the 10 highest intensity parent ions (MS2 scan). The resolution of the second-order mass spectrometry was 17500 @ m/z 200, AGV target: 1e5, and the second-order Maximum IT:50 ms, MS2 Activation Type: HCD, Isolation window:2 m/z, normalized collision energy (Setpped): 10, 20, 30.

### Data preprocessing

The original data were calibrated by MSDIAL software for peak alignment, retention time correction and peak area extraction. The structure of metabolites was identified by accurate mass number matching (mass deviation masstolerance < 20ppm) and secondary spectrum matching (mass deviation mass tolerance < 0.02Da). Public databases such as HMDB, MassBank and our self-built metabolite standard database were searched. For the extracted data, deleting the ion peaks with missing values > 50% in the group did not participate in the follow-up statistical analysis; normalizing the total peak area of the positive and negative ion data respectively, integrating the positive and negative ion peaks and using R software for pattern recognition, the data were preprocessed by Unit variance scaling (UV), and the follow-up data were analyzed.

## Bioinformatic analysis

The global proteins and metabolites changes data in the hippocampus were analyzed with the Perseus software, Microsoft Excel and R statistical computing software. In the analysis of significant difference of protein, the method of T test (Student's t test) binding multiple of change (Fold change, FC, the ratio of average expression quantity between the two groups) was used to screen the significant difference protein (*P*-value < 0.05 and FC > 1.1 or < 0.91 at the same time). The selection of differential metabolites had both multivariate statistical analysis (VIP > 1) and univariate statistical analysis (*P*-value < 0.05 and FC > 1.5 or < 0.67). GO and KEGG enrichment analysis were analyzed by Fisher accurate test, and multiple tests were corrected by FDR. The construction of protein-protein interaction (PPI) network is also carried out by using String database of cytoscape software. In all the analyses, p < 0.05 was considered to be statistically significant.

# Supplementary Figures and Tables

## Supplementary Tables

**Table S1. Identified Significant differences in hippocampal proteins in the diabetic mice.**

| NO | Accession | Gene Symbol | Protein Name | MW [kDa] | DM/CC | P value |
| --- | --- | --- | --- | --- | --- | --- |
| 1 | P62071 | Rras2 | Ras-related protein R-Ras2 | 23.4 | 1.62 | 0.0412 |
| 2 | Q61016 | Gng7 | Guanine nucleotide-binding protein G(I)/G(S)/G(O) subunit gamma-7 | 7.5 | 1.59 | 0.0208 |
| 3 | Q8BFU2 | H2aw | Histone H2A type 3 | 14.1 | 1.58 | 0.0064 |
| 4 | A0A087WQG6 | Rgs6 | Regulator of G-protein-signaling 6 | 53.8 | 1.47 | 0.0205 |
| 5 | A0A0N4SW28 | Gng12 | Guanine nucleotide-binding protein subunit gamma | 9.2 | 1.46 | 0.0214 |
| 6 | P51910 | Apod | Apolipoprotein D | 21.5 | 1.41 | 0.0118 |
| 7 | Q9JMF3 | Gng13 | Guanine nucleotide-binding protein G(I)/G(S)/G(O) subunit gamma-13 | 8 | 1.35 | 0.0192 |
| 8 | Q9CPQ1 | Cox6c | Cytochrome c oxidase subunit 6C | 8.5 | 1.34 | 0.0018 |
| 9 | P48771 | Cox7a2 | Cytochrome c oxidase subunit 7A2, mitochondrial | 9.3 | 1.33 | 0.0008 |
| 10 | P62311 | Lsm3 | U6 snRNA-associated Sm-like protein LSm3 | 11.8 | 1.31 | 0.0163 |
| 11 | O09114 | Ptgds | Prostaglandin-H2 D-isomerase | 21.1 | 1.31 | 0.0055 |
| 12 | A0A654ICL5 | Gjc3 | Gap junction protein | 29 | 1.30 | 0.0456 |
| 13 | P24549 | Aldh1a1 | Aldehyde dehydrogenase 1A1 | 54.4 | 1.30 | 0.0231 |
| 14 | Q03958 | Pfdn6 | Prefoldin subunit 6 | 14.4 | 1.30 | 0.0032 |
| 15 | P17665 | Cox7c | Cytochrome c oxidase subunit 7C, mitochondrial | 7.3 | 1.28 | 0.0024 |
| 16 | Q9D0K2 | Oxct1 | Succinyl-CoA:3-ketoacid coenzyme A transferase 1, mitochondrial | 56 | 1.28 | 0.0152 |
| 17 | D3YWY6 | Mpc1 | Mitochondrial pyruvate carrier | 9.7 | 1.27 | 0.0041 |
| 18 | A2A4P3 | Gm27029 | Predicted gene, 27029 | 58.1 | 1.26 | 0.0326 |
| 19 | B1AXP6 | Tomm5 | Mitochondrial import receptor subunit TOM5 homolog | 6 | 1.26 | 0.0108 |
| 20 | A0A088DEG2 | Shank3 | Shank3e splice variant IV | 18.2 | 1.24 | 0.0046 |
| 21 | Q9JJL8 | Sars2 | Serine--tRNA ligase, mitochondrial | 58.3 | 1.24 | 0.0075 |
| 22 | Q9Z2Q6 | Septin5 | Septin-5 | 42.7 | 1.24 | 0.0125 |
| 23 | P97822 | Anp32e | Acidic leucine-rich nuclear phosphoprotein 32 family member E | 29.6 | 1.23 | 0.0240 |
| 24 | Q06185 | Atp5k | ATP synthase subunit e, mitochondrial | 8.2 | 1.22 | 0.0158 |
| 25 | A2BDX3 | Mocs3 | Adenylyl transferase and sulfur transferase MOCS3 | 49.3 | 1.22 | 0.0017 |
| 26 | Q9WU84 | Ccs | Copper chaperone for superoxide dismutase | 28.9 | 1.21 | 0.0083 |
| 27 | Q04447 | Ckb | Creatine kinase B-type | 42.7 | 1.21 | 0.0087 |
| 28 | Q9DBG5 | Plin3 | Perilipin-3 | 47.2 | 1.20 | 0.0037 |
| 29 | Q80ZJ7 | Snx32 | Sorting nexin-32 | 46.6 | 1.20 | 0.0000 |
| 30 | A0A571BEC9 | Plin4 | Perilipin-4 | 158.6 | 1.19 | 0.0095 |
| 31 | A0A1L1SQX6 | Olfm2 | Noelin-2 | 51.6 | 1.19 | 0.0419 |
| 32 | Q9Z2I8 | Suclg2 | Succinate--CoA ligase [GDP-forming] subunit beta, mitochondrial | 46.8 | 1.19 | 0.0035 |
| 33 | Q62348 | Tsn | Translin | 26.2 | 1.18 | 0.0001 |
| 34 | Q3TZP3 | Mta2 | Uncharacterized protein | 75 | 1.18 | 0.0009 |
| 35 | Q8BTD8 | Pitpnc1 | Uncharacterized protein (Fragment) | 39.2 | 1.18 | 0.0179 |
| 36 | Q91VZ6 | Smap1 | Stromal membrane-associated protein 1 | 47.6 | 1.18 | 0.0224 |
| 37 | Q9ERE8 | Tlnrd1 | Talin rod domain-containing protein 1 | 37.8 | 1.18 | 0.0136 |
| 38 | Q8C0C9 | Tspan2 | Tetraspanin (Fragment) | 21.6 | 1.18 | 0.0460 |
| 39 | Q8R0W0 | Eppk1 | Epiplakin | 724.2 | 1.18 | 0.0041 |
| 40 | Q99JT1 | Gatb | Glutamyl-tRNA (Gln) amidotransferase subunit B, mitochondrial | 62.1 | 1.18 | 0.0029 |
| 41 | B0QZN5 | Vamp2 | Synaptobrevin-2 | 17.9 | 1.17 | 0.0329 |
| 42 | Q9CZW4 | Acsl3 | Fatty acid CoA ligase Acsl3 | 80.4 | 1.17 | 0.0009 |
| 43 | Q9CZC8 | Scrn1 | Secernin-1 | 46.3 | 1.17 | 0.0142 |
| 44 | P63216 | Gng3 | Guanine nucleotide-binding protein G(I)/G(S)/G(O) subunit gamma-3 | 8.3 | 1.17 | 0.0074 |
| 45 | O89086 | Rbm3 | RNA-binding protein 3 | 16.6 | 1.17 | 0.0304 |
| 46 | P17809 | Slc2a1 | Solute carrier family 2, facilitated glucose transporter member 1 | 53.9 | 1.17 | 0.0022 |
| 47 | Q05D93 | Rufy2 | Rufy2 protein (Fragment) | 45.9 | 1.17 | 0.0006 |
| 48 | B1AZ39 | Chmp6 | Chromatin modifying protein 6 | 23.4 | 1.16 | 0.0359 |
| 49 | A0A068BIT0 | Slc39a7 | Solute carrier family 39 | 50.6 | 1.16 | 0.0054 |
| 50 | Q3U9G9 | Lbr | Delta (14)-sterol reductase LBR | 71.4 | 1.16 | 0.0080 |
| 51 | Q8VDN2 | Atp1a1 | Sodium/potassium-transporting ATPase subunit alpha-1 | 112.9 | 1.16 | 0.0473 |
| 52 | Q99L13 | Hibadh | 3-hydroxyisobutyrate dehydrogenase, mitochondrial | 35.4 | 1.16 | 0.0016 |
| 53 | Q9DCB1 | Hmgn3 | High mobility group nucleosome-binding domain-containing protein 3 | 10.8 | 1.16 | 0.0106 |
| 54 | Q0VEW4 | Plp2 | Proteolipid protein 2 | 16.6 | 1.16 | 0.0362 |
| 55 | E9Q3J1 | Dtx3 | E3 ubiquitin-protein ligase | 42.9 | 1.15 | 0.0146 |
| 56 | Q9JL56 | Gde1 | Glycerophosphodiester phosphodiesterase 1 | 37.6 | 1.15 | 0.0068 |
| 57 | P15105 | Glul | Glutamine synthetase | 42.1 | 1.15 | 0.0022 |
| 58 | Q5NCJ9 | Uqcr10 | Complex III subunit 9 | 7.4 | 1.15 | 0.0313 |
| 59 | Q3UTZ3 | Trappc14 | Trafficking protein particle complex subunit 14 | 62.7 | 1.15 | 0.0258 |
| 60 | Q8BGR3 | Camk4 | Calcium/calmodulin-dependent protein kinase IV | 52.5 | 1.15 | 0.0036 |
| 61 | A0A0U1RNS6 | Hddc3 | Guanosine-3',5'-bis(diphosphate) 3'-pyrophosphohydrolase MESH1 | 16.1 | 1.15 | 0.0234 |
| 62 | Q3UDA8 | Cpt2 | Carn_acyltransf domain-containing protein | 73.9 | 1.15 | 0.0003 |
| 63 | Q9DBM1 | Gpatch1 | G patch domain-containing protein 1 | 102.9 | 1.15 | 0.0016 |
| 64 | A2AJQ1 | Dpy19l4 | Probable C-mannosyl transferase DPY19L4 (Fragment) | 60.7 | 1.15 | 0.0355 |
| 65 | Q68FG3 | Spty2d1 | Protein SPT2 homolog | 74.8 | 1.15 | 0.0051 |
| 66 | Q9JIX8 | Acin1 | Apoptotic chromatin condensation inducer in the nucleus | 150.6 | 1.15 | 0.0017 |
| 67 | A0A0A6YVR9 | Surf1 | SURF1-like protein (Fragment) | 27.1 | 1.15 | 0.0148 |
| 68 | P68372 | Tubb4b | Tubulin beta-4B chain | 49.8 | 1.15 | 0.0133 |
| 69 | Q9CQU0 | Txndc12 | Thioredoxin domain-containing protein 12 | 19 | 1.15 | 0.0057 |
| 70 | F8WGX5 | Dmxl1 | DmX-like protein 1 | 337.2 | 1.14 | 0.0021 |
| 71 | Q9QZE7 | Tsnax | Translin-associated protein X | 32.9 | 1.14 | 0.0033 |
| 72 | Q3UYV9 | Ncbp1 | Nuclear cap-binding protein subunit 1 | 91.9 | 1.14 | 0.0260 |
| 73 | P39038 | Cdh4 | Cadherin-4 | 100 | 1.14 | 0.0078 |
| 74 | Q8BH50 | 8030462N17Rik | Uncharacterized protein C18orf25 homolog | 26.4 | 1.14 | 0.0219 |
| 75 | P63137 | Gabrb2 | Gamma-aminobutyric acid receptor subunit beta-2 | 59.2 | 1.14 | 0.0242 |
| 76 | Q9D3U7 | Macroh2a3 | Histone H2A | 22.5 | 1.14 | 0.0167 |
| 77 | E9Q827 | Arpp19 | cAMP-regulated phosphoprotein 19 | 16.1 | 1.14 | 0.0091 |
| 78 | P28650 | Adssl1 | Adenylosuccinate synthetase isozyme 1 | 50.2 | 1.14 | 0.0297 |
| 79 | Q790Y8 | G6pdx | Glucose-6-phosphate 1-dehydrogenase | 59.2 | 1.14 | 0.0098 |
| 80 | A2ALS7 | Rap1gap | Rap1 GTPase-activating protein 1 (Fragment) | 43.4 | 1.14 | 0.0212 |
| 81 | Q67BT3 | Slc13a5 | Solute carrier family 13-member 5 | 63.8 | 1.13 | 0.0244 |
| 82 | Q9D846 | Ndufc2 | NADH dehydrogenase [ubiquinone] 1 subunit C2 | 14.3 | 1.13 | 0.0105 |
| 83 | Q80TE2 | Pcdh10 | MKIAA1400 protein (Fragment) | 119.6 | 1.13 | 0.0049 |
| 84 | P53995 | Anapc1 | Anaphase-promoting complex subunit 1 | 215.9 | 1.13 | 0.0487 |
| 85 | P14152 | Mdh1 | Malate dehydrogenase, cytoplasmic | 36.5 | 1.13 | 0.0459 |
| 86 | Q8CBE3 | Wdr37 | WD repeat-containing protein 37 | 55 | 1.13 | 0.0226 |
| 87 | Q9WUR2 | Eci2 | Enoyl-CoA delta isomerase 2 | 43.2 | 1.13 | 0.0185 |
| 88 | P61079 | Ube2d3 | Ubiquitin-conjugating enzyme E2 D3 | 16.7 | 1.13 | 0.0003 |
| 89 | H3BJM1 | Foxp1 | Forkhead box protein P1 | 75.1 | 1.13 | 0.0027 |
| 90 | Q8QZV4 | Stk32c | Serine/threonine-protein kinase 32C | 55.2 | 1.13 | 0.0280 |
| 91 | Q921M7 | Cyrib | CYFIP-related Rac1 interactor B | 36.8 | 1.12 | 0.0487 |
| 92 | Q9JHS9 | Cwc15 | Spliceosome-associated protein CWC15 homolog | 26.6 | 1.12 | 0.0070 |
| 93 | Q9DCG9 | Trmt112 | Multifunctional methyltransferase subunit TRM112-like protein | 14.1 | 1.12 | 0.0251 |
| 94 | Q3UP61 | Dlg1 | Uncharacterized protein | 96.7 | 1.12 | 0.0369 |
| 95 | Q80XN0 | Bdh1 | D-beta-hydroxybutyrate dehydrogenase, mitochondrial | 38.3 | 1.12 | 0.0220 |
| 96 | O35621 | Pmm1 | Phosphomannomutase 1 | 29.8 | 1.12 | 0.0158 |
| 97 | A0A087WRY3 | Nucks1 | Nuclear ubiquitous casein and cyclin-dependent kinase substrate 1 | 26.2 | 1.12 | 0.0298 |
| 98 | P09528 | Fth1 | Ferritin heavy chain | 21.1 | 1.12 | 0.0301 |
| 99 | Q62425 | Ndufa4 | Cytochrome c oxidase subunit NDUFA4 | 9.3 | 1.12 | 0.0041 |
| 100 | E9QKR0 | Gnb2 | Guanine nucleotide-binding protein G(I)/G(S)/G(T) subunit beta-2 | 41.4 | 1.12 | 0.0242 |
| 101 | Q8K1A5 | Tmem41b | Transmembrane protein 41B | 32.4 | 1.12 | 0.0052 |
| 102 | A2ANL1 | Slc23a2 | Solute carrier family 23 member 2 (Fragment) | 4.3 | 1.12 | 0.0454 |
| 103 | A0A6I8MX27 | Ldhb | L-lactate dehydrogenase | 37.3 | 1.12 | 0.0303 |
| 104 | Q9D3P8 | Plgrkt | Plasminogen receptor (KT) | 17.3 | 1.12 | 0.0103 |
| 105 | Q5KU39 | Vps41 | Vacuolar protein sorting-associated protein 41 homolog | 98.5 | 1.12 | 0.0032 |
| 106 | Q3V3R1 | Mthfd1l | Monofunctional C1-tetrahydrofolate synthase, mitochondrial | 105.7 | 1.12 | 0.0103 |
| 107 | Q8VEB6 | Elac1 | Zinc phosphodiesterase ELAC protein 1 | 39.7 | 1.12 | 0.0313 |
| 108 | Q3TMB8 | Adsl | Adenylosuccinate lyase | 54.8 | 1.12 | 0.0003 |
| 109 | Q91WT9 | Cbs | Cystathionine beta-synthase | 61.5 | 1.12 | 0.0131 |
| 110 | Q9WU79 | Prodh | Proline dehydrogenase 1, mitochondrial | 68 | 1.12 | 0.0163 |
| 111 | Q8BS91 | Son | Uncharacterized protein (Fragment) | 59.2 | 1.12 | 0.0302 |
| 112 | P0DN34 | Ndufb1 | NADH dehydrogenase [ubiquinone] 1 beta subcomplex subunit 1 | 7 | 1.12 | 0.0041 |
| 113 | A0A023J6F3 | COX2 | Cytochrome c oxidase subunit 2 | 26 | 1.12 | 0.0004 |
| 114 | A0A0R4J008 | Hdac2 | Histone deacetylase 2 | 55.3 | 1.12 | 0.0282 |
| 115 | Q3UTI7 | Pin1 | Peptidyl-prolyl cis-trans isomerase | 18.4 | 1.11 | 0.0112 |
| 116 | Q62095 | Ddx3y | ATP-dependent RNA helicase DDX3Y | 73.4 | 1.11 | 0.0062 |
| 117 | Q4ZJM7 | Otol1 | Otolin-1 | 49.6 | 1.11 | 0.0209 |
| 118 | Q8BJZ4 | Mrps35 | 28S ribosomal protein S35, mitochondrial | 36 | 1.11 | 0.0192 |
| 119 | O08579 | Emd | Emerin | 29.4 | 1.11 | 0.0226 |
| 120 | Q80SY6 | Adal | Adenosine deaminase-like protein | 41 | 1.11 | 0.0413 |
| 121 | E9Q7U2 | Calcoco1 | Calcium-binding and coiled-coil domain-containing protein 1 | 70.9 | 1.11 | 0.0057 |
| 122 | Q8BNN1 | Spata2l | Spermatogenesis-associated protein 2-like protein | 46.7 | 1.11 | 0.0097 |
| 123 | Q8R366 | Igsf8 | Immunoglobulin superfamily member 8 | 65 | 1.11 | 0.0219 |
| 124 | A2A8Y5 | Oscp1 | Organic solute carrier 1 (Fragment) | 43.2 | 1.11 | 0.0196 |
| 125 | Q8BMS1 | Hadha | Trifunctional enzyme subunit alpha, mitochondrial | 82.6 | 1.11 | 0.0007 |
| 126 | Q5F2D0 | Acsl6 | Uncharacterized protein | 80.7 | 1.11 | 0.0056 |
| 127 | O08599 | Stxbp1 | Syntaxin-binding protein 1 | 67.5 | 1.11 | 0.0105 |
| 128 | Q9DCL9 | Paics | Multifunctional protein ADE2 | 47 | 1.11 | 0.0055 |
| 129 | Q3TIJ4 | Rtca | RNA 3'-terminal phosphate cyclase | 39.3 | 1.11 | 0.0161 |
| 130 | P19783 | Cox4i1 | Cytochrome c oxidase subunit 4 isoform 1, mitochondrial | 19.5 | 1.11 | 0.0033 |
| 131 | B2RQF5 | Mdga2 | Mdga2 protein | 107.4 | 1.11 | 0.0020 |
| 132 | G3UZY2 | Txn2 | Thioredoxin, mitochondrial (Fragment) | 14.2 | 1.11 | 0.0396 |
| 133 | A0A0R4J023 | Auh | Methylglutaconyl-CoA hydratase, mitochondrial | 33.3 | 1.11 | 0.0319 |
| 134 | B2RWI2 | Lactb | Lactamase, beta | 60.7 | 1.11 | 0.0291 |
| 135 | Q3TV47 | Atp1b1 | Sodium/potassium-transporting ATPase subunit beta | 35.2 | 1.11 | 0.0148 |
| 136 | Q8K1H1 | Tdrd7 | Tudor domain-containing protein 7 | 122.1 | 1.11 | 0.0315 |
| 137 | Q9CX86 | Hnrnpa0 | Heterogeneous nuclear ribonucleoprotein A0 | 30.5 | 1.10 | 0.0404 |
| 138 | Q8VEH8 | Erlec1 | Endoplasmic reticulum lectin 1 | 54.9 | 1.10 | 0.0195 |
| 139 | Q7TPW6 | Slc25a20 | Solute carrier family 25 (Mitochondrial carnitine/acylcarnitine translocase), member 20 | 32.9 | 1.10 | 0.0101 |
| 140 | Q540D7 | Akr1a1 | Alcohol dehydrogenase [NADP (+)] | 36.6 | 1.10 | 0.0028 |
| 141 | Q9DB20 | Atp5o | ATP synthase subunit O, mitochondrial | 23.3 | 1.10 | 0.0161 |
| 142 | P62075 | Timm13 | Mitochondrial import inner membrane translocase subunit Tim13 | 10.5 | 1.10 | 0.0448 |
| 143 | P12787 | Cox5a | Cytochrome c oxidase subunit 5A, mitochondrial | 16.1 | 1.10 | 0.0220 |
| 144 | Q9CQD1 | Rab5a | Ras-related protein Rab-5A | 23.6 | 1.10 | 0.0398 |
| 145 | Q99LC3 | Ndufa10 | NADH dehydrogenase [ubiquinone] 1 alpha subcomplex subunit 10, mitochondrial | 40.6 | 1.10 | 0.0009 |
| 146 | Q3UEL5 | Uroc1 | Imidazolonepropionate hydrolase | 78.4 | 1.10 | 0.0468 |
| 147 | P61264 | Stx1b | Syntaxin-1B | 33.2 | 1.10 | 0.0064 |
| 148 | Q99K70 | Rragc | Ras-related GTP-binding protein C | 44.1 | 0.91 | 0.0148 |
| 149 | O35685 | Nudc | Nuclear migration protein nudC | 38.3 | 0.91 | 0.0004 |
| 150 | E9PZ43 | Map4 | Microtubule-associated protein | 97.7 | 0.91 | 0.0421 |
| 151 | E9PYG6 | Rasa1 | RAS p21 protein activator 1 | 115.4 | 0.91 | 0.0118 |
| 152 | P63017 | Hspa8 | Heat shock cognate 71 kDa protein | 70.8 | 0.91 | 0.0095 |
| 153 | F6XC25 | Cc2d1b | Coiled-coil and C2 domain-containing protein 1B (Fragment) | 85 | 0.91 | 0.0286 |
| 154 | Q3U8D2 | St13 | STI1 domain-containing protein | 41.5 | 0.91 | 0.0151 |
| 155 | Q6PCX7 | Rgma | Repulsive guidance molecule A | 49.6 | 0.91 | 0.0215 |
| 156 | Q99LM2 | Cdk5rap3 | CDK5 regulatory subunit-associated protein 3 | 57 | 0.91 | 0.0293 |
| 157 | A2AWS5 | Hdac5 | Histone deacetylase | 111.4 | 0.91 | 0.0207 |
| 158 | Q922R8 | Pdia6 | Protein disulfide-isomerase A6 | 48.1 | 0.91 | 0.0479 |
| 159 | A2AVJ7 | Rrbp1 | Ribosome-binding protein 1 | 158.3 | 0.91 | 0.0272 |
| 160 | Q9CVB6 | Arpc2 | Actin-related protein 2/3 complex subunit 2 | 34.3 | 0.91 | 0.0467 |
| 161 | Q149L6 | Dnajb14 | DnaJ homolog subfamily B member 14 | 42.3 | 0.90 | 0.0129 |
| 162 | Q99L48 | Nmd3 | 60S ribosomal export protein NMD3 | 57.6 | 0.90 | 0.0347 |
| 163 | Q9WUT3 | Rps6ka2 | Ribosomal protein S6 kinase alpha-2 | 83.1 | 0.90 | 0.0456 |
| 164 | B0FTY3 | Nudcd3 | NudC-like protein | 40.9 | 0.90 | 0.0077 |
| 165 | Q2KHK7 | Mal2 | Mal, T-cell differentiation protein 2 | 19.1 | 0.90 | 0.0134 |
| 166 | A0A1D5RMG4 | Agap3 | Arf-GAP with GTPase, ANK repeat and PH domain-containing protein 3 | 42.4 | 0.90 | 0.0440 |
| 167 | P62259 | Ywhae | 14-3-3 protein epsilon | 29.2 | 0.90 | 0.0114 |
| 168 | Q8BQ46 | Taf15 | TAF15 RNA polymerase II, TATA box binding protein (TBP)-associated factor | 58.6 | 0.90 | 0.0426 |
| 169 | Q8C7H1 | Mmaa | Methylmalonic aciduria type A homolog, mitochondrial | 45.9 | 0.90 | 0.0176 |
| 170 | Q9DBP5 | Cmpk1 | UMP-CMP kinase | 22.2 | 0.90 | 0.0022 |
| 171 | Q9EQQ9 | Oga | Protein O-GlcNAcase | 103.1 | 0.90 | 0.0096 |
| 172 | B3V097 | Cstf2 | BetaCstF-64 variant 3 | 66.4 | 0.90 | 0.0015 |
| 173 | Q7TQ95 | Lnpk | Endoplasmic reticulum junction formation protein lunapark | 47.5 | 0.90 | 0.0124 |
| 174 | Q3U8S0 | Ap3s1 | Adaptor-related protein complex 3, sigma 1 subunit | 21.7 | 0.90 | 0.0156 |
| 175 | Q60936 | Coq8a | Atypical kinase COQ8A, mitochondrial | 71.7 | 0.90 | 0.0024 |
| 176 | A8IP69 | Ywhag | 14-3-3 protein gamma subtype | 28.3 | 0.90 | 0.0055 |
| 177 | Q8K0C9 | Gmds | GDP-mannose 4,6 dehydratase | 42 | 0.90 | 0.0245 |
| 178 | Q80UL3 | Galk1 | Galactokinase 1 | 42.3 | 0.90 | 0.0023 |
| 179 | P48024 | Eif1 | Eukaryotic translation initiation factor 1 | 12.7 | 0.90 | 0.0121 |
| 180 | Q9CQV4 | Retreg3 | Reticulophagy regulator 3 | 51.6 | 0.90 | 0.0283 |
| 181 | Q6VNS1 | Ntrk3 | NT-3 growth factor receptor | 92.7 | 0.90 | 0.0300 |
| 182 | Q80X87 | Gmfb | Glia maturation factor | 16.8 | 0.90 | 0.0454 |
| 183 | A2AS45 | Pkp4 | Plakophilin-4 | 128.4 | 0.89 | 0.0332 |
| 184 | Q8C0L9 | Gpcpd1 | Glycerophosphocholine phosphodiesterase GPCPD1 | 76.5 | 0.89 | 0.0040 |
| 185 | P61961 | Ufm1 | Ubiquitin-fold modifier 1 | 9.1 | 0.89 | 0.0051 |
| 186 | Q8R570 | Snap47 | Synaptosomal-associated protein 47 | 46.5 | 0.89 | 0.0032 |
| 187 | P51655 | Gpc4 | Glypican-4 | 62.5 | 0.89 | 0.0405 |
| 188 | A0A0R4J138 | Arsb | Arylsulfatase B | 59.7 | 0.89 | 0.0353 |
| 189 | A0A0R4J1R7 | Pcbd2 | 4a-hydroxytetrahydrobiopterin dehydratase | 11.7 | 0.89 | 0.0464 |
| 190 | Q8BHE3 | Atcay | Caytaxin | 42.2 | 0.89 | 0.0043 |
| 191 | Q62418 | Dbnl | Drebrin-like protein | 48.7 | 0.89 | 0.0261 |
| 192 | B2RXU5 | Zfp365 | Zfp365 protein | 46.8 | 0.89 | 0.0083 |
| 193 | P47867 | Scg3 | Secretogranin-3 | 53.3 | 0.89 | 0.0476 |
| 194 | Q3UVU3 | Slc30a10 | Zinc transporter 10 | 50.9 | 0.89 | 0.0078 |
| 195 | J3QNA0 | Ntng2 | Netrin-G2 | 60 | 0.89 | 0.0216 |
| 196 | Q3TXB1 | C1qa | Complement C1q subcomponent subunit A | 26 | 0.89 | 0.0117 |
| 197 | Q8CH77 | Nav1 | Neuron navigator 1 | 202.2 | 0.89 | 0.0394 |
| 198 | A0A087WSM1 | Ica1l | Islet cell autoantigen 1-like protein | 49.1 | 0.89 | 0.0056 |
| 199 | P14206 | Rpsa | 40S ribosomal protein SA | 32.8 | 0.89 | 0.0148 |
| 200 | Q8BHC4 | Dcakd | Dephospho-CoA kinase domain-containing protein | 26.5 | 0.89 | 0.0126 |
| 201 | Q3TWF2 | Hspa5 | 78 kDa glucose-regulated protein | 72.4 | 0.89 | 0.0336 |
| 202 | Q9CR16 | Ppid | Peptidyl-prolyl cis-trans isomerase D | 40.7 | 0.89 | 0.0186 |
| 203 | A0A5F8MPL9 | Dock9 | Dedicator of cytokinesis protein 9 | 235.2 | 0.89 | 0.0139 |
| 204 | Q7TSH6 | Scaf4 | SR-related and CTD-associated factor 4 | 129 | 0.89 | 0.0116 |
| 205 | D3Y273 | Slc4a7 | Anion exchange protein | 123.2 | 0.89 | 0.0470 |
| 206 | B0LAE3 | Ptn | Pleiotrophin (Fragment) | 7.7 | 0.89 | 0.0459 |
| 207 | Q3TDI5 | Pex19 | Peroxin-19 | 32.7 | 0.89 | 0.0015 |
| 208 | D3Z6I8 | Tpm3 | Tropomyosin alpha-3 chain | 28.7 | 0.89 | 0.0043 |
| 209 | Q4FJN9 | Fdps | Fdps protein | 40.6 | 0.89 | 0.0074 |
| 210 | Q6PHU5 | Sort1 | Sortilin | 91.1 | 0.89 | 0.0388 |
| 211 | E9PYH2 | Acot7 | Cytosolic acyl coenzyme A thioester hydrolase | 42.8 | 0.89 | 0.0014 |
| 212 | Q8C129 | Lnpep | Leucyl-cystinyl aminopeptidase | 117.2 | 0.89 | 0.0167 |
| 213 | P47915 | Rpl29 | 60S ribosomal protein L29 | 17.6 | 0.89 | 0.0391 |
| 214 | Q60900 | Elavl3 | ELAV-like protein 3 | 39.5 | 0.88 | 0.0233 |
| 215 | E9Q452 | Tpm1 | Tropomyosin alpha-1 chain | 32.5 | 0.88 | 0.0426 |
| 216 | G3X972 | Sec24c | Sec24-related gene family, member C (S. cerevisiae) | 118.5 | 0.88 | 0.0023 |
| 217 | Q5DU37 | Zfyve26 | Zinc finger FYVE domain-containing protein 26 | 282.8 | 0.88 | 0.0030 |
| 218 | Q9CQV8 | Ywhab | 14-3-3 protein beta/alpha | 28.1 | 0.88 | 0.0189 |
| 219 | Q9QUP5 | Hapln1 | Hyaluronan and proteoglycan link protein 1 | 40.5 | 0.88 | 0.0372 |
| 220 | Q62048 | Pea15a | Astrocytic phosphoprotein PEA-15 | 15 | 0.88 | 0.0228 |
| 221 | B2MWM9 | Calr | Calreticulin | 48 | 0.88 | 0.0025 |
| 222 | P12382 | Pfkl | ATP-dependent 6-phosphofructokinase, liver type | 85.3 | 0.88 | 0.0068 |
| 223 | Z4YJT3 | Larp1 | La-related protein 1 | 121.1 | 0.88 | 0.0061 |
| 224 | Q61699 | Hsph1 | Heat shock protein 105 kDa | 96.3 | 0.88 | 0.0028 |
| 225 | Q99JG2 | Gpr37l1 | G-protein coupled receptor 37-like 1 | 52.7 | 0.88 | 0.0086 |
| 226 | Q60864 | Stip1 | Stress-induced-phosphoprotein 1 | 62.5 | 0.88 | 0.0169 |
| 227 | Q8BL86 | Mblac2 | Acyl-coenzyme A thioesterase MBLAC2 | 31.2 | 0.88 | 0.0145 |
| 228 | Q9Z0R9 | Fads2 | Acyl-CoA 6-desaturase | 52.4 | 0.88 | 0.0418 |
| 229 | A0A140LHW9 | Rab30 | Ras-related protein Rab-30 (Fragment) | 12.2 | 0.88 | 0.0019 |
| 230 | Q7TSY6 | Celf4 | CUGBP Elav-like family member 4 | 51.9 | 0.88 | 0.0238 |
| 231 | Q9CYG7 | Tomm34 | Mitochondrial import receptor subunit TOM34 | 34.3 | 0.88 | 0.0081 |
| 232 | D3Z4B0 | Srsf11 | Serine and arginine-rich-splicing factor 11 (Fragment) | 19.3 | 0.88 | 0.0179 |
| 233 | Q4KL76 | Hspe1 | 10 kDa heat shock protein, mitochondrial | 11 | 0.88 | 0.0092 |
| 234 | A0A087WRH2 | Faim | Fas apoptotic inhibitory molecule 1 (Fragment) | 19.6 | 0.88 | 0.0322 |
| 235 | Q9CX80 | Cygb | Cytoglobin | 21.5 | 0.88 | 0.0046 |
| 236 | F6T4M4 | Srrm1 | Serine/arginine repetitive matrix protein 1 (Fragment) | 16.2 | 0.87 | 0.0287 |
| 237 | Q8BI84 | Mia3 | Transport and Golgi organization protein 1 homolog | 213.5 | 0.87 | 0.0013 |
| 238 | Q6ZWQ6 | Ube2k | E2 ubiquitin-conjugating enzyme | 22.4 | 0.87 | 0.0323 |
| 239 | Q9WVE8 | Pacsin2 | Protein kinase C and casein kinase substrate in neurons protein 2 | 55.8 | 0.87 | 0.0138 |
| 240 | Q9R0P3 | Esd | S-formylglutathione hydrolase | 31.3 | 0.87 | 0.0052 |
| 241 | Q9D554 | Sf3a3 | Splicing factor 3A subunit 3 | 58.8 | 0.87 | 0.0488 |
| 242 | Q8K187 | Cybrd1 | Cytochrome b reductase 1 (Fragment) | 15.4 | 0.87 | 0.0303 |
| 243 | P24472 | Gsta4 | Glutathione S-transferase A4 | 25.5 | 0.87 | 0.0185 |
| 244 | Q3UHL1 | Camkv | CaM kinase-like vesicle-associated protein | 54.8 | 0.87 | 0.0138 |
| 245 | Q3YAA9 | Kcnip3 | Calsenilin isoform 4 | 26.4 | 0.87 | 0.0028 |
| 246 | Q6PGE7 | Slc6a7 | Sodium-dependent proline transporter | 71 | 0.87 | 0.0132 |
| 247 | Q6P9R5 | Gnptab | Gnptab protein | 104.9 | 0.87 | 0.0182 |
| 248 | B2KF50 | Uhrf1bp1 | UHRF1 (ICBP90)-binding protein 1 | 156.9 | 0.86 | 0.0190 |
| 249 | Q8VDH1 | Fbxo21 | F-box only protein 21 | 72.1 | 0.86 | 0.0118 |
| 250 | Q9QYR9 | Acot2 | Acyl-coenzyme A thioesterase 2, mitochondrial | 49.6 | 0.86 | 0.0036 |
| 251 | Q9D4I9 | Rab23 | Ras-related protein Rab-23 | 26.8 | 0.86 | 0.0031 |
| 252 | A0A0R4J0W7 | Lmtk3 | Serine/threonine-protein kinase LMTK3 | 150.8 | 0.86 | 0.0324 |
| 253 | A0A7U3JW65 | Fgf12 | Fibroblast growth factor | 27.4 | 0.86 | 0.0006 |
| 254 | Q3UM52 |  | Polypeptide N-acetylgalactosaminyltransferase (Fragment) | 69.4 | 0.86 | 0.0033 |
| 255 | Q5SX50 | Pfn1 | Profilin | 14.9 | 0.86 | 0.0110 |
| 256 | Q8R3Q1 | F3 | Tissue factor | 32.9 | 0.86 | 0.0352 |
| 257 | E9Q3K3 | Agap1 | Arf-GAP with GTPase, ANK repeat and PH domain-containing protein 1 | 115.7 | 0.86 | 0.0145 |
| 258 | P07759 | Serpina3k | Serine protease inhibitor A3K | 46.9 | 0.86 | 0.0498 |
| 259 | Q8BJU0 | Sgta | Small glutamine-rich tetratricopeptide repeat-containing protein alpha | 34.3 | 0.85 | 0.0119 |
| 260 | Q9D7X1 | Kctd4 | BTB/POZ domain-containing protein KCTD4 | 30 | 0.85 | 0.0301 |
| 261 | K3W4L3 | Psap | Prosaposin | 61.3 | 0.85 | 0.0491 |
| 262 | Q920P5 | Ak5 | Adenylate kinase isoenzyme 5 | 63.3 | 0.85 | 0.0253 |
| 263 | P05532 | Kit | Mast/stem cell growth factor receptor Kit | 109.3 | 0.85 | 0.0237 |
| 264 | P83940 | Eloc | Elongin-C | 12.5 | 0.85 | 0.0079 |
| 265 | A0A0G2JGN4 | Snrpb | Small nuclear ribonucleoprotein-associated protein B | 8.4 | 0.85 | 0.0377 |
| 266 | Q9CPW4 | Arpc5 | Actin-related protein 2/3 complex subunit 5 | 16.3 | 0.85 | 0.0215 |
| 267 | Q99J49 | Tubb2a | Tubb2a protein (Fragment) | 34 | 0.85 | 0.0158 |
| 268 | Q3TCN2 | Plbd2 | Putative phospholipase B-like 2 | 66.2 | 0.84 | 0.0266 |
| 269 | Q8R0Y8 | Slc25a42 | Mitochondrial coenzyme A transporter SLC25A42 | 35.2 | 0.84 | 0.0026 |
| 270 | Q3U4G8 | Coro2a | Coronin | 59.6 | 0.84 | 0.0358 |
| 271 | E9QK20 | Cacna1e | Voltage-dependent R-type calcium channel subunit alpha | 223 | 0.84 | 0.0148 |
| 272 | Q3UUI3 | Them4 | Acyl-coenzyme A thioesterase THEM4 | 26 | 0.84 | 0.0181 |
| 273 | Q14BZ3 | Lxn | Latexin | 25.5 | 0.84 | 0.0164 |
| 274 | A2AWA9 | Rabgap1 | Rab GTPase-activating protein 1 | 120.7 | 0.84 | 0.0040 |
| 275 | E9Q0H6 | Fabp7 | Fatty acid-binding protein, brain | 20.6 | 0.84 | 0.0040 |
| 276 | Q9Z1N5 | Ddx39b | Spliceosome RNA helicase Ddx39b | 49 | 0.84 | 0.0162 |
| 277 | Q8C5P7 | Tdrp | Testis development-related protein | 20.2 | 0.84 | 0.0472 |
| 278 | Q6A078 | Cep290 | Centrosomal protein of 290 kDa | 288.9 | 0.83 | 0.0077 |
| 279 | P07901 | Hsp90aa1 | Heat shock protein HSP 90-alpha | 84.7 | 0.83 | 0.0071 |
| 280 | Q99LT0 | Dpy30 | Protein dpy-30 homolog | 11.2 | 0.83 | 0.0284 |
| 281 | Q52L78 | Cryab | Alpha(B)-crystallin | 20.1 | 0.83 | 0.0190 |
| 282 | P03995 | Gfap | Glial fibrillary acidic protein | 49.9 | 0.83 | 0.0116 |
| 283 | Q78JE5 | Fbxo22 | F-box only protein 22 | 44.2 | 0.83 | 0.0017 |
| 284 | Q05A62 | Dnal1 | Dynein axonemal light chain 1 | 21.5 | 0.83 | 0.0021 |
| 285 | P61211 | Arl1 | ADP-ribosylation factor-like protein 1 | 20.4 | 0.83 | 0.0017 |
| 286 | Q3V3U7 | Zer1 | Uncharacterized protein | 40.8 | 0.83 | 0.0137 |
| 287 | Q9Z140 | Cpne6 | Copine-6 | 61.7 | 0.83 | 0.0483 |
| 288 | Q14A12 | Rac3 | RAS-related C3 botulinum substrate 3 | 21.4 | 0.82 | 0.0254 |
| 289 | Q9QY42 | Gpr37 | Prosaposin receptor GPR37 | 66.7 | 0.82 | 0.0128 |
| 290 | P34884 | Mif | Macrophage migration inhibitory factor | 12.5 | 0.82 | 0.0358 |
| 291 | Q8C413 | Dgkg | Diacylglycerol kinase | 83.9 | 0.81 | 0.0481 |
| 292 | Q546G4 | Alb | Serum albumin | 68.6 | 0.81 | 0.0275 |
| 293 | Q71LX8 | Hsp90ab1 | Heat shock protein 84b | 83.2 | 0.81 | 0.0027 |
| 294 | Q6P3D0 | Nudt16 | U8 snoRNA-decapping enzyme | 21.8 | 0.81 | 0.0181 |
| 295 | Q9D1P4 | Chordc1 | Cysteine and histidine-rich domain-containing protein 1 | 37.3 | 0.80 | 0.0013 |
| 296 | B2B9E1 | Triqk | Triple QxxK/R motif-containing protein | 9.7 | 0.80 | 0.0049 |
| 297 | Q8BGZ1 | Hpcal4 | Hippocalcin-like protein 4 | 22.2 | 0.80 | 0.0079 |
| 298 | F8WHU6 | Cdh9 | Cadherin-9 | 88.5 | 0.80 | 0.0265 |
| 299 | Q8VCN9 | Tbcc | Tubulin-specific chaperone C | 38.1 | 0.80 | 0.0316 |
| 300 | Q8BVI5 | Stx16 | Syntaxin-16 | 37.1 | 0.80 | 0.0173 |
| 301 | Q3UZV3 | Pcsk2 | P/Homo B domain-containing protein | 68.9 | 0.80 | 0.0463 |
| 302 | P62774 | Mtpn | Myotrophin | 12.9 | 0.79 | 0.0054 |
| 303 | Q5M9N6 | Rpl37a | Rpl37a protein | 9 | 0.79 | 0.0290 |
| 304 | D3YTQ3 | Hnrnpdl | Heterogeneous nuclear ribonucleoprotein D-like | 46.2 | 0.79 | 0.0055 |
| 305 | A0A0A6YWP9 | Txndc9 | Thioredoxin domain-containing protein 9 (Fragment) | 19.7 | 0.78 | 0.0121 |
| 306 | Q9JHZ2 | Ank | Progressive ankylosis protein | 54.3 | 0.78 | 0.0304 |
| 307 | Q9DBS2 | Tprgl | Tumor protein p63-regulated gene 1-like protein | 29.8 | 0.78 | 0.0153 |
| 308 | F6RCM5 | Necap2 | Adaptin ear-binding coat-associated protein 2 (Fragment) | 8.8 | 0.78 | 0.0103 |
| 309 | Q9R1B9 | Slit2 | Slit homolog 2 protein | 168.7 | 0.78 | 0.0085 |
| 310 | Q3UPX0 | Crym | Ketimine reductase mu-crystallin | 33.5 | 0.78 | 0.0075 |
| 311 | Q6A028 | Swap70 | Switch-associated protein 70 | 69 | 0.78 | 0.0106 |
| 312 | Q8R1Y2 | Bmerb1 | bMERB domain-containing protein 1 | 23.5 | 0.77 | 0.0454 |
| 313 | A5GZX3 | Glo1 | Lactoylglutathione lyase | 20.8 | 0.77 | 0.0271 |
| 314 | K7X735 | ND2 | NADH-ubiquinone oxidoreductase chain 2 | 38.8 | 0.77 | 0.0227 |
| 315 | Q9JK28 | Ncapg | Uncharacterized protein (Fragment) | 32.5 | 0.76 | 0.0170 |
| 316 | Q6P7T7 | Rab2b | Rab2b protein | 8.8 | 0.76 | 0.0007 |
| 317 | P63154 | Crnkl1 | Crooked neck-like protein 1 | 83.4 | 0.75 | 0.0157 |
| 318 | Q7TN73 | Casd1 | N-acetylneuraminate 9-O-acetyltransferase | 91.5 | 0.75 | 0.0187 |
| 319 | Q9JJV2 | Pfn2 | Profilin-2 | 15 | 0.75 | 0.0008 |
| 320 | Q5DTR1 | Rapgef6 | MKIAA4052 protein (Fragment) | 116.3 | 0.75 | 0.0123 |
| 321 | A0A286T4B5 | COX1 | Cytochrome c oxidase subunit 1 | 56.9 | 0.74 | 0.0122 |
| 322 | Q80UH6 | Nod2 | NOD2 | 112.7 | 0.74 | 0.0038 |
| 323 | Q8R3W2 | 0610009B22Rik | RIKEN cDNA 0610009B22 gene | 16.4 | 0.73 | 0.0177 |
| 324 | Q8R2S3 | Nol3 | Nucleolar protein 3 (Apoptosis repressor with CARD domain) | 24.6 | 0.73 | 0.0004 |
| 325 | Q63810 | Ppp3r1 | Calcineurin subunit B type 1 | 19.3 | 0.72 | 0.0205 |
| 326 | Q8QZT2 | Ccsap | Centriole, cilia and spindle-associated protein | 28.4 | 0.70 | 0.0373 |
| 327 | Q3UT71 | A630010A05Rik | Uncharacterized protein (Fragment) | 26.5 | 0.67 | 0.0240 |
| 328 | Q99PE9 | Arl4d | ADP-ribosylation factor-like protein 4D | 22.3 | 0.67 | 0.0110 |
| 329 | Q9CPU4 | Mgst3 | Microsomal glutathione S-transferase 3 | 16.9 | 0.52 | 0.0006 |

**Table S2. Identified Significant differences in hippocampal proteins in diabetic mice as reversed by D-pinitol.**

| NO | Accession | Gene Symbol | Protein Name | MW [kDa] | DP/DM | P value |
| --- | --- | --- | --- | --- | --- | --- |
| 1 | Q04447 | Ckb | Creatine kinase B-type | 42.7 | 0.88 | 0.0174 |
| 2 | A0A6I8MX27 | Ldhb | L-lactate dehydrogenase | 37.3 | 0.90 | 0.0306 |
| 3 | Q9Z2Q6 | Septin5 | Septin-5 | 42.7 | 0.82 | 0.0326 |
| 4 | Q9CZC8 | Scrn1 | Secernin-1 | 46.3 | 0.86 | 0.0152 |
| 5 | Q3UP61 | Dlg1 | Uncharacterized protein | 96.7 | 0.87 | 0.0216 |
| 6 | Q8BGR3 | Camk4 | Calcium/calmodulin-dependent protein kinase IV | 52.5 | 0.91 | 0.0094 |
| 7 | Q99L13 | Hibadh | 3-hydroxyisobutyrate dehydrogenase, mitochondrial | 35.4 | 0.88 | 0.0028 |
| 8 | P12787 | Cox5a | Cytochrome c oxidase subunit 5A, mitochondrial | 16.1 | 0.87 | 0.0124 |
| 9 | Q4ACU6 | Shank3 | SH3 and multiple ankyrin repeat domains protein 3 | 185.3 | 0.91 | 0.0335 |
| 10 | A2ALS7 | Rap1gap | Rap1 GTPase-activating protein 1 (Fragment) | 43.4 | 0.90 | 0.0455 |
| 11 | P24549 | Aldh1a1 | Aldehyde dehydrogenase 1A1 | 54.4 | 0.73 | 0.0051 |
| 12 | E9Q827 | Arpp19 | cAMP-regulated phosphoprotein 19 | 16.1 | 0.82 | 0.0023 |
| 13 | Q06185 | Atp5k | ATP synthase subunit e, mitochondrial | 8.2 | 0.85 | 0.0419 |
| 14 | Q9CPQ1 | Cox6c | Cytochrome c oxidase subunit 6C | 8.5 | 0.77 | 0.0340 |
| 15 | A0A087WQG6 | Rgs6 | Regulator of G-protein-signaling 6 | 53.8 | 0.83 | 0.0353 |
| 16 | A2A4P3 | Gm27029 | Predicted gene, 27029 | 58.1 | 0.75 | 0.0327 |
| 17 | Q80TE2 | Pcdh10 | MKIAA1400 protein (Fragment) | 119.6 | 0.89 | 0.0093 |
| 18 | Q3TZP3 | Mta2 | Uncharacterized protein | 75 | 0.86 | 0.0197 |
| 19 | P62311 | Lsm3 | U6 snRNA-associated Sm-like protein LSm3 | 11.8 | 0.83 | 0.0324 |
| 20 | Q3UTZ3 | Trappc14 | Trafficking protein particle complex subunit 14 | 62.7 | 0.80 | 0.0177 |
| 21 | Q8BTD8 | Pitpnc1 | Uncharacterized protein (Fragment) | 39.2 | 0.90 | 0.0454 |
| 22 | Q9DBG5 | Plin3 | Perilipin-3 | 47.2 | 0.86 | 0.0320 |
| 23 | Q0VEW4 | Plp2 | Proteolipid protein 2 | 16.6 | 0.82 | 0.0094 |
| 24 | Q68FG3 | Spty2d1 | Protein SPT2 homolog | 74.8 | 0.87 | 0.0283 |
| 25 | Q99JT1 | Gatb | Glutamyl-tRNA (Gln) amidotransferase subunit B, mitochondrial | 62.1 | 0.91 | 0.0492 |
| 26 | Q9ERE8 | Tlnrd1 | Talin rod domain-containing protein 1 | 37.8 | 0.85 | 0.0108 |
| 27 | Q99J49 | Tubb2a | Tubb2a protein (Fragment) | 34 | 1.17 | 0.0145 |
| 28 | Q9CQV8 | Ywhab | 14-3-3 protein beta/alpha | 28.1 | 1.16 | 0.0063 |
| 29 | Q9Z140 | Cpne6 | Copine-6 | 61.7 | 1.15 | 0.0042 |
| 30 | Q5SX50 | Pfn1 | Profilin | 14.9 | 1.15 | 0.0231 |
| 31 | Q9JJV2 | Pfn2 | Profilin-2 | 15 | 1.31 | 0.0006 |
| 32 | P14206 | Rpsa | 40S ribosomal protein SA | 32.8 | 1.13 | 0.0051 |
| 33 | Q9CPW4 | Arpc5 | Actin-related protein 2/3 complex subunit 5 | 16.3 | 1.13 | 0.0031 |
| 34 | P62774 | Mtpn | Myotrophin | 12.9 | 1.34 | 0.0413 |
| 35 | Q8C413 | Dgkg | Diacylglycerol kinase | 83.9 | 1.21 | 0.0461 |
| 36 | D3YTQ3 | Hnrnpdl | Heterogeneous nuclear ribonucleoprotein D-like | 46.2 | 1.36 | 0.0012 |
| 37 | Q9QYR9 | Acot2 | Acyl-coenzyme A thioesterase 2, mitochondrial | 49.6 | 1.15 | 0.0166 |
| 38 | Q9Z1N5 | Ddx39b | Spliceosome RNA helicase Ddx39b | 49 | 1.24 | 0.0115 |
| 39 | P24472 | Gsta4 | Glutathione S-transferase A4 | 25.5 | 1.10 | 0.0316 |
| 40 | A2AWA9 | Rabgap1 | Rab GTPase-activating protein 1 | 120.7 | 1.10 | 0.0491 |
| 41 | Q9R0P3 | Esd | S-formylglutathione hydrolase | 31.3 | 1.17 | 0.0247 |
| 42 | Q8BL86 | Mblac2 | Acyl-coenzyme A thioesterase MBLAC2 | 31.2 | 1.12 | 0.0057 |
| 43 | A0A0R4J0W7 | Lmtk3 | Serine/threonine-protein kinase LMTK3 | 150.8 | 1.18 | 0.0111 |
| 44 | Q6P7T7 | Rab2b | Rab2b protein | 8.8 | 1.31 | 0.0002 |
| 45 | Q14BZ3 | Lxn | Latexin | 25.5 | 1.18 | 0.0022 |
| 46 | Q9D4I9 | Rab23 | Ras-related protein Rab-23 | 26.8 | 1.21 | 0.0341 |
| 47 | Q9WUT3 | Rps6ka2 | Ribosomal protein S6 kinase alpha-2 | 83.1 | 1.14 | 0.0179 |
| 48 | Q05A62 | Dnal1 | Dynein axonemal light chain 1 | 21.5 | 1.10 | 0.0056 |
| 49 | Q7TSY6 | Celf4 | CUGBP Elav-like family member 4 | 51.9 | 1.11 | 0.0141 |
| 50 | Q5DTR1 | Rapgef6 | MKIAA4052 protein (Fragment) | 116.3 | 1.35 | 0.0028 |
| 51 | Q6PGE7 | Slc6a7 | Sodium-dependent proline transporter | 71 | 1.13 | 0.0222 |
| 52 | Q78JE5 | Fbxo22 | F-box only protein 22 | 44.2 | 1.24 | 0.0032 |
| 53 | P63154 | Crnkl1 | Crooked neck-like protein 1 | 83.4 | 1.30 | 0.0330 |
| 54 | Q9CPU4 | Mgst3 | Microsomal glutathione S-transferase 3 | 16.9 | 1.81 | 0.0002 |
| 55 | Q9D7X1 | Kctd4 | BTB/POZ domain-containing protein KCTD4 | 30 | 1.12 | 0.0032 |
| 56 | A0A7U3JW65 | Fgf12 | Fibroblast growth factor | 27.4 | 1.15 | 0.0018 |
| 57 | Q6PCX7 | Rgma | Repulsive guidance molecule A | 49.6 | 1.19 | 0.0080 |
| 58 | Q8VDH1 | Fbxo21 | F-box only protein 21 | 72.1 | 1.18 | 0.0051 |
| 59 | Q7TSH6 | Scaf4 | SR-related and CTD-associated factor 4 | 129 | 1.16 | 0.0046 |
| 60 | A0A0G2JGN4 | Snrpb | Small nuclear ribonucleoprotein-associated protein B | 8.4 | 1.23 | 0.0159 |
| 61 | Q8K187 | Cybrd1 | Cytochrome b reductase 1 (Fragment) | 15.4 | 1.19 | 0.0031 |
| 62 | Q7TN73 | Casd1 | N-acetylneuraminate 9-O-acetyltransferase | 91.5 | 1.38 | 0.0066 |
| 63 | Q9DBS2 | Tprgl | Tumor protein p63-regulated gene 1-like protein | 29.8 | 1.15 | 0.0276 |
| 64 | Q8R0Y8 | Slc25a42 | Mitochondrial coenzyme A transporter SLC25A42 | 35.2 | 1.16 | 0.0121 |
| 65 | Q5M9N6 | Rpl37a | Rpl37a protein | 9 | 1.44 | 0.0428 |
| 66 | Q6A078 | Cep290 | Centrosomal protein of 290 kDa | 288.9 | 1.16 | 0.0008 |
| 67 | A2AWS5 | Hdac5 | Histone deacetylase | 111.4 | 1.14 | 0.0218 |
| 68 | A0A0A6YWP9 | Txndc9 | Thioredoxin domain-containing protein 9 (Fragment) | 19.7 | 1.20 | 0.0037 |
| 69 | Q9R1B9 | Slit2 | Slit homolog 2 protein | 168.7 | 1.33 | 0.0105 |
| 70 | Q2KHK7 | Mal2 | Mal, T-cell differentiation protein 2 | 19.1 | 1.16 | 0.0352 |
| 71 | B2RXU5 | Zfp365 | Zfp365 protein | 46.8 | 1.14 | 0.0100 |
| 72 | Q99PE9 | Arl4d | ADP-ribosylation factor-like protein 4D | 22.3 | 1.48 | 0.0006 |

**Table S3. Identified significant differences in hippocampal metabolites in the diabetic mice.**

| NO | MetaboName | KEGGID | Super Class | DM/CC | VIP | P.value |
| --- | --- | --- | --- | --- | --- | --- |
| 1 | Waikialoid A |  | --- | 149.51 | 1.73 | 0.0001 |
| 2 | Melatonin | C01598 | Organ heterocyclic compounds | 72.25 | 1.13 | 0.0275 |
| 3 | Nomegestrol Acetate |  | --- | 28.89 | 1.05 | 0.0482 |
| 4 | Naproxen | C01517 | Benzenoids | 16.45 | 1.09 | 0.0498 |
| 5 | S-Adenosyl-L-methionine | C00019 | Nucleosides, nucleotides, and analogues | 15.52 | 2.01 | 0.0000 |
| 6 | Veratramine | C10829 | --- | 15.12 | 1.18 | 0.0246 |
| 7 | Dehydroabietamide |  | --- | 14.34 | 1.11 | 0.0369 |
| 8 | 3-Methyl-2-(2-Nonen-1-Yl)-4-Quinolinol |  | --- | 10.56 | 1.08 | 0.0415 |
| 9 | Ouabain | C01443 | Lipids and lipid-like molecules | 10.39 | 1.98 | 0.0000 |
| 10 | Nomifensine Maleate Salt |  | --- | 8.67 | 1.08 | 0.0433 |
| 11 | 3-O-Methylfunicone |  | --- | 8.48 | 1.47 | 0.0009 |
| 12 | Phenyltoloxamine Citrate |  | --- | 7.97 | 1.07 | 0.0457 |
| 13 | Formononetin | C00858 | Phenylpropanoids and polyketides | 7.48 | 1.33 | 0.0020 |
| 14 | Arenobufagin | C20035 | --- | 7.03 | 2.02 | 0.0000 |
| 15 | Thymoquinone |  | Organic oxygen compounds | 6.07 | 1.67 | 0.0001 |
| 16 | Bisoprolol | C06852 | Benzenoids | 5.83 | 1.63 | 0.0005 |
| 17 | Laccaic Acid A |  | --- | 5.38 | 1.24 | 0.0165 |
| 18 | Sulfapyridine |  | Benzenoids | 5.18 | 1.95 | 0.0000 |
| 19 | N,N`-Dimethyl-N,N`-Diphenylurea |  | --- | 5.12 | 1.47 | 0.0024 |
| 20 | Evodiamine | C09187 | --- | 4.92 | 2.08 | 0.0000 |
| 21 | 4-Methylabyssinone V |  | --- | 4.64 | 1.51 | 0.0025 |
| 22 | Indole-3-Acetyl-L-Valine |  | --- | 4.61 | 2.21 | 0.0000 |
| 23 | Calycanthine | C10573 | Organ heterocyclic compounds | 4.60 | 2.04 | 0.0000 |
| 24 | Toddalolactone |  | --- | 4.60 | 2.14 | 0.0000 |
| 25 | Vanillin | C00755 | Benzenoids | 3.82 | 2.11 | 0.0000 |
| 26 | Kurarinone | C17446 | --- | 3.81 | 1.96 | 0.0000 |
| 27 | 1-Naphthonitrile |  | --- | 3.73 | 2.03 | 0.0000 |
| 28 | N-Cyclohexyl-2-Benzothiazol-Amine |  | Organ heterocyclic compounds | 3.62 | 1.30 | 0.0107 |
| 29 | Acetaminophen Glucuronide |  | Organic oxygen compounds | 3.55 | 1.37 | 0.0027 |
| 30 | Cinnamoylcholine |  | --- | 3.50 | 2.05 | 0.0000 |
| 31 | Ticlopidine Hydrochloride |  | Organ heterocyclic compounds | 3.43 | 1.55 | 0.0011 |
| 32 | Quinidine | C06527 | Alkaloids and derivatives | 3.39 | 1.95 | 0.0000 |
| 33 | Geranylgeranyl 1,4-Hydroquinone |  | --- | 3.16 | 1.97 | 0.0000 |
| 34 | Arctiin | C16915 | --- | 3.11 | 1.28 | 0.0118 |
| 35 | Sebuthylazine |  | --- | 3.07 | 2.08 | 0.0000 |
| 36 | Geodin Hydrate |  | --- | 2.89 | 1.20 | 0.0101 |
| 37 | Hirsutine | C16972 | --- | 2.87 | 2.16 | 0.0000 |
| 38 | Kushenol I |  | --- | 2.75 | 1.83 | 0.0000 |
| 39 | Piperlotine A |  | --- | 2.63 | 2.22 | 0.0000 |
| 40 | Pinacidil Monohydrate |  | --- | 2.58 | 1.47 | 0.0026 |
| 41 | Triacetin | D00384 | Lipids and lipid-like molecules | 2.56 | 2.03 | 0.0000 |
| 42 | 6-Ethoxy-2-Mercaptobenzothiazole |  | Organ heterocyclic compounds | 2.55 | 1.12 | 0.0310 |
| 43 | 8-Prenylnaringenin | C18023 | --- | 2.53 | 1.82 | 0.0000 |
| 44 | Loureirin A |  | --- | 2.51 | 2.01 | 0.0000 |
| 45 | 5-Hydroxymethylcytidine |  | Organ heterocyclic compounds | 2.51 | 2.06 | 0.0000 |
| 46 | Gigantol |  | --- | 2.48 | 1.89 | 0.0000 |
| 47 | Wedelolactone | C10541 | --- | 2.45 | 1.51 | 0.0020 |
| 48 | Glabrone |  | Phenylpropanoids and polyketides | 2.42 | 1.44 | 0.0010 |
| 49 | Sesamin | C10882 | Lignans, neolignanes and related compounds | 2.35 | 1.21 | 0.0067 |
| 50 | (5-L-Glutamyl)-L-glutamine | C05283 | Organic acids and derivatives | 2.34 | 2.20 | 0.0000 |
| 51 | Celogentin C |  | --- | 2.31 | 1.05 | 0.0165 |
| 52 | Cholecalciferol | C05443 | Lipids and lipid-like molecules | 2.26 | 1.09 | 0.0399 |
| 53 | Urolithin B |  | Phenylpropanoids and polyketides | 2.23 | 1.65 | 0.0003 |
| 54 | 1,2-Dihydroxyheptadec-16-Yn-4-Yl Acetate |  | --- | 2.22 | 1.33 | 0.0130 |
| 55 | Caffeoylcholine |  | --- | 2.21 | 1.62 | 0.0005 |
| 56 | Ala-Ile |  | Organic acids and derivatives | 2.17 | 2.16 | 0.0000 |
| 57 | Purpurogallin-4-Carboxylic Acid |  | --- | 2.14 | 1.34 | 0.0122 |
| 58 | Taspine |  | --- | 2.13 | 1.78 | 0.0000 |
| 59 | Pymetrozine | C18590 | --- | 2.10 | 2.03 | 0.0000 |
| 60 | D-Mannose-6-Phosphate | C00275 | --- | 2.08 | 1.42 | 0.0033 |
| 61 | 3-Amino-3-(4-Hydroxyphenyl) Propionic Acid |  | Organic acids and derivatives | 2.06 | 1.35 | 0.0086 |
| 62 | 1-Naphthol | C11714 | Benzenoids | 2.06 | 2.04 | 0.0000 |
| 63 | 6-Hydroxybenzofuran-3(2H)-One |  | --- | 1.99 | 2.20 | 0.0000 |
| 64 | Thymine | C00178 | Organ heterocyclic compounds | 1.98 | 2.06 | 0.0000 |
| 65 | 4-(Sec-Butoxy) Benzoic Acid |  | --- | 1.94 | 1.65 | 0.0002 |
| 66 | Pantothenic Acid | C00864 | Organic oxygen compounds | 1.94 | 1.99 | 0.0000 |
| 67 | 2-(3,4-Dimethoxyphenyl)-7-Methoxy-4H-Chromen-4-One |  | --- | 1.93 | 1.81 | 0.0000 |
| 68 | Anethole | C10428 | Benzenoids | 1.92 | 2.16 | 0.0000 |
| 69 | L-5-Oxoproline | C01879 | Organic acids and derivatives | 1.91 | 2.14 | 0.0000 |
| 70 | Lychnopholide |  | --- | 1.90 | 1.73 | 0.0000 |
| 71 | 4-Acetylbutyric Acid | C02129 | Lipids and lipid-like molecules | 1.89 | 1.90 | 0.0000 |
| 72 | Benzyl Isothiocyanate | C03098 | Benzenoids | 1.89 | 1.60 | 0.0002 |
| 73 | Maltol | C11918 | Organ heterocyclic compounds | 1.88 | 1.05 | 0.0269 |
| 74 | 5,6-Dihydro-5-Methyluracil | C00906 | Organ heterocyclic compounds | 1.87 | 2.08 | 0.0000 |
| 75 | N, N-Dimethylsulfamide |  | --- | 1.87 | 1.99 | 0.0000 |
| 76 | Glutamine | C00303 | Organic acids and derivatives | 1.87 | 2.17 | 0.0000 |
| 77 | Tigogenin | C08914 | Lipids and lipid-like molecules | 1.86 | 1.80 | 0.0000 |
| 78 | 4-Hydroxymandelonitrile | C00650 | Benzenoids | 1.83 | 1.34 | 0.0096 |
| 79 | cis-4-Hydroxy-L-proline | C01015 | Organic acids and derivatives | 1.83 | 1.27 | 0.0045 |
| 80 | 7,8-Dimethylalloxazine | C01727 | --- | 1.80 | 1.18 | 0.0192 |
| 81 | Avocadyne Acetate |  | --- | 1.79 | 1.76 | 0.0001 |
| 82 | Genipin 1-Gentiobioside |  | --- | 1.79 | 1.23 | 0.0184 |
| 83 | Pyridoxal | C00250 | Organ heterocyclic compounds | 1.78 | 1.26 | 0.0167 |
| 84 | 1,3-Diaminopyrene |  | Organ heterocyclic compounds | 1.78 | 2.09 | 0.0000 |
| 85 | Gyromitrin | C08305 | Organic nitrogen compounds | 1.77 | 2.16 | 0.0000 |
| 86 | Orbencarb | C11087 | --- | 1.76 | 1.59 | 0.0007 |
| 87 | Agnus ide | C09765 | Lipids and lipid-like molecules | 1.75 | 1.35 | 0.0072 |
| 88 | Theobromine | C07480 | Organ heterocyclic compounds | 1.74 | 1.69 | 0.0000 |
| 89 | Nor cyclobenzaprine |  | --- | 1.73 | 2.06 | 0.0000 |
| 90 | Lycorine | C08532 | --- | 1.72 | 1.63 | 0.0005 |
| 91 | D-Alanyl-D-alanine | C00993 | Organic acids and derivatives | 1.70 | 2.00 | 0.0000 |
| 92 | Erotic Acid | C00295 | Organ heterocyclic compounds | 1.69 | 1.97 | 0.0000 |
| 93 | Panaxydiol |  | --- | 1.68 | 1.60 | 0.0001 |
| 94 | Eremanthin | C09406 | --- | 1.68 | 1.97 | 0.0000 |
| 95 | Gluconasturtiin | C08417 | Organic oxygen compounds | 1.67 | 1.24 | 0.0186 |
| 96 | L- (+)-Arginine | C00062 | Organic acids and derivatives | 1.66 | 1.82 | 0.0000 |
| 97 | Glutamine (L) |  | --- | 1.66 | 1.50 | 0.0022 |
| 98 | Carnosine | C00386 | Organic acids and derivatives | 1.66 | 1.83 | 0.0001 |
| 99 | OCP |  | Organ heterocyclic compounds | 1.63 | 1.17 | 0.0159 |
| 100 | 3,4,5-Trimethoxycinnamic Acid |  | Phenylpropanoids and polyketides | 1.62 | 1.59 | 0.0004 |
| 101 | Trigonelline | C01004 | Alkaloids and derivatives | 1.61 | 2.07 | 0.0000 |
| 102 | Linoleoyl Ethanolamide |  | Organic nitrogen compounds | 1.60 | 1.84 | 0.0000 |
| 103 | Diferuloyl Putrescine |  | --- | 1.59 | 1.16 | 0.0061 |
| 104 | Trans-Cinnamaldehyde | C00903 | Phenylpropanoids and polyketides | 1.59 | 1.36 | 0.0053 |
| 105 | cis-Aconitate | C00417 | Organic acids and derivatives | 1.56 | 1.35 | 0.0075 |
| 106 | Acetoacetate | C00164 | Organic acids and derivatives | 1.51 | 1.77 | 0.0000 |
| 107 | DDAO |  | --- | 1.51 | 1.20 | 0.0201 |
| 108 | L-Methionine Sulfone |  | Organic acids and derivatives | 1.50 | 1.50 | 0.0003 |
| 109 | 2-Methylanthra-9,10-Quinone |  | --- | 0.03 | 2.09 | 0.0000 |
| 110 | Deoxyguanylic Acid | C00362 | Nucleosides, nucleotides, and analogues | 0.16 | 1.38 | 0.0014 |
| 111 | L-Saccharopine | C00449 | Organic acids and derivatives | 0.25 | 2.09 | 0.0000 |
| 112 | Canthin-6-One | C09098 | --- | 0.27 | 1.43 | 0.0058 |
| 113 | Sarafloxacin HCl |  | --- | 0.27 | 1.62 | 0.0007 |
| 114 | Dyclonine Hydrochloride |  | Organic oxygen compounds | 0.31 | 1.47 | 0.0021 |
| 115 | Diuron | C18428 | --- | 0.33 | 1.82 | 0.0000 |
| 116 | Pinoxaden |  | --- | 0.33 | 1.28 | 0.0090 |
| 117 | Mannitol | C00392 | Organic oxygen compounds | 0.35 | 1.83 | 0.0000 |
| 118 | (5E)-Heptadeca-5,16-Diene-1,2,4-Triol |  | --- | 0.36 | 1.71 | 0.0002 |
| 119 | 3- (Alpha, Alpha-Dimethylallyl)Psoralen | C09165 | --- | 0.37 | 1.83 | 0.0000 |
| 120 | Aloenin |  | --- | 0.38 | 2.03 | 0.0000 |
| 121 | NADH | C00004 | Nucleosides, nucleotides, and analogues | 0.40 | 1.50 | 0.0005 |
| 122 | Avicularin |  | Phenylpropanoids and polyketides | 0.40 | 1.50 | 0.0011 |
| 123 | Microcolin E |  | --- | 0.41 | 2.00 | 0.0000 |
| 124 | Malonic Acid | C00383 | Organic acids and derivatives | 0.41 | 1.95 | 0.0000 |
| 125 | Beta-Sitosterol | C01753 | Lipids and lipid-like molecules | 0.42 | 1.18 | 0.0286 |
| 126 | Luteolin | C01514 | Phenylpropanoids and polyketides | 0.42 | 1.64 | 0.0001 |
| 127 | Ibutilide Fumarate |  | Benzenoids | 0.42 | 1.44 | 0.0012 |
| 128 | Citric Acid | C00158 | Organic acids and derivatives | 0.42 | 1.89 | 0.0000 |
| 129 | Debromoaplysiatoxin | C05148 | --- | 0.43 | 1.51 | 0.0003 |
| 130 | Chloro-IB-MECA |  | --- | 0.45 | 1.61 | 0.0007 |
| 131 | Epigallocatechin 3-gallate | C09731 | Phenylpropanoids and polyketides | 0.45 | 1.16 | 0.0328 |
| 132 | N-Benzyloleamide |  | --- | 0.46 | 1.52 | 0.0014 |
| 133 | 3-Methylhistidine | C01152 | Organic acids and derivatives | 0.47 | 1.60 | 0.0012 |
| 134 | 3-Hexadecyl-4-Methoxy-5-Methyloxolan-2-One |  | --- | 0.48 | 1.08 | 0.0489 |
| 135 | N-Acetyl-Asp-Glu |  | --- | 0.48 | 2.08 | 0.0000 |
| 136 | 2-Hydroxyacetanilide |  | Benzenoids | 0.49 | 1.67 | 0.0001 |
| 137 | Bromperidol |  | --- | 0.49 | 2.06 | 0.0000 |
| 138 | Prosulfocarb | C18760 | --- | 0.49 | 1.45 | 0.0008 |
| 139 | 1-(2-(1H-Indol-3-Yl)Ethyl)Urea |  | --- | 0.50 | 1.93 | 0.0000 |
| 140 | Coptisine | C16938 | --- | 0.51 | 1.33 | 0.0027 |
| 141 | Butyryl Carnitine |  | --- | 0.51 | 2.01 | 0.0000 |
| 142 | Syringic Acid | C10833 | Benzenoids | 0.51 | 1.34 | 0.0047 |
| 143 | Inosine 5'-monophosphate | C00130 | Nucleosides, nucleotides, and analogues | 0.53 | 1.51 | 0.0020 |
| 144 | Prenylamine Lactate |  | --- | 0.53 | 1.53 | 0.0021 |
| 145 | Rosmanol |  | --- | 0.53 | 2.05 | 0.0000 |
| 146 | Miglitol | C07708 | Organoheterocyclic compounds | 0.53 | 1.66 | 0.0001 |
| 147 | 1-Acetylimidazole |  | --- | 0.54 | 1.89 | 0.0000 |
| 148 | Nicotinic Acid Mono Nucleotide |  | --- | 0.55 | 1.46 | 0.0028 |
| 149 | Bisanhydrorutilantinone |  | --- | 0.55 | 1.23 | 0.0110 |
| 150 | 2-Ureidopentanedioic Acid |  | --- | 0.55 | 1.44 | 0.0010 |
| 151 | Methyl Orsellinate |  | --- | 0.55 | 1.46 | 0.0005 |
| 152 | Uridine 5'-Monophosphate | C00105 | Nucleosides, nucleotides, and analogues | 0.56 | 1.66 | 0.0004 |
| 153 | 1,3,6-Tri-O-Methylarthothelin |  | --- | 0.57 | 1.93 | 0.0000 |
| 154 | Tonantzitlolone B |  | --- | 0.57 | 1.33 | 0.0158 |
| 155 | Isodolichantoside |  | --- | 0.57 | 1.47 | 0.0012 |
| 156 | Scytonemin |  | --- | 0.57 | 1.27 | 0.0150 |
| 157 | 5-Aminovaleric Acid Betaine |  | Organoheterocyclic compounds | 0.58 | 1.74 | 0.0001 |
| 158 | 2-Morpholinothiobenzothiazole |  | --- | 0.58 | 1.37 | 0.0026 |
| 159 | 6-Hydroxysumatrol |  | --- | 0.59 | 1.92 | 0.0000 |
| 160 | 3-Phosphonopropionic Acid |  | --- | 0.59 | 1.47 | 0.0023 |
| 161 | Protodioscin | C08907 | Lipids and lipid-like molecules | 0.59 | 1.72 | 0.0003 |
| 162 | 1-Methyladenosine | C02494 | Nucleosides, nucleotides, and analogues | 0.59 | 2.04 | 0.0000 |
| 163 | Oxypeucedanin Hydrate |  | --- | 0.59 | 2.05 | 0.0000 |
| 164 | Propionic Acid | C00163 | Organic acids and derivatives | 0.60 | 1.82 | 0.0000 |
| 165 | Roemerine |  | Organoheterocyclic compounds | 0.60 | 1.88 | 0.0000 |
| 166 | Fenoldopam | C07693 | Organoheterocyclic compounds | 0.61 | 1.76 | 0.0002 |
| 167 | Isofraxidin | C17480 | --- | 0.61 | 1.66 | 0.0002 |
| 168 | Meloxicam | C08169 | Organ heterocyclic compounds | 0.61 | 1.21 | 0.0213 |
| 169 | Gamma-Mangostin |  | Organ heterocyclic compounds | 0.61 | 1.75 | 0.0000 |
| 170 | O-Phospho-L-serine | C01005 | Organic acids and derivatives | 0.61 | 1.21 | 0.0129 |
| 171 | Fluanisone |  | --- | 0.61 | 1.25 | 0.0036 |
| 172 | L-Argininosuccinate | C03406 | Organic acids and derivatives | 0.61 | 1.86 | 0.0000 |
| 173 | Phenanthraquinone | C03243 | --- | 0.61 | 1.49 | 0.0009 |
| 174 | Calcimycin | C11309 | --- | 0.62 | 1.58 | 0.0012 |
| 175 | Alpha-Ergocryptine | C07545 | --- | 0.62 | 1.14 | 0.0338 |
| 176 | Berberrubine |  | Alkaloids and derivatives | 0.62 | 1.20 | 0.0192 |
| 177 | Heliocurassavicine N-Oxyde |  | --- | 0.62 | 2.01 | 0.0000 |
| 178 | Arabinofuranosyluracil |  | --- | 0.62 | 1.77 | 0.0000 |
| 179 | Dl-Lauroylcarnitine |  | --- | 0.63 | 1.82 | 0.0000 |
| 180 | Apotoxicarol |  | --- | 0.63 | 1.47 | 0.0032 |
| 181 | (+)-Bicuculline | C09364 | --- | 0.63 | 1.33 | 0.0124 |
| 182 | 8-Gingerol |  | --- | 0.63 | 1.24 | 0.0042 |
| 183 | Aztreonam | C06840 | Organ heterocyclic compounds | 0.63 | 1.30 | 0.0055 |
| 184 | 8-Shogaol |  | Benzenoids | 0.63 | 1.60 | 0.0001 |
| 185 | Polygalic Acid |  | --- | 0.64 | 1.96 | 0.0000 |
| 186 | Carnitine | C00487 | Organic nitrogen compounds | 0.64 | 1.99 | 0.0000 |
| 187 | Caffeic Acid | C01481 | Phenylpropanoids and polyketides | 0.64 | 1.27 | 0.0037 |
| 188 | Riboflavin | C00255 | Nucleosides, nucleotides, and analogues | 0.64 | 1.53 | 0.0003 |
| 189 | Mevalonic Acid Lactone |  | Organ heterocyclic compounds | 0.64 | 1.46 | 0.0044 |
| 190 | Hdmboa |  | Organ heterocyclic compounds | 0.65 | 1.24 | 0.0103 |
| 191 | Conchacarpine A |  | --- | 0.65 | 1.52 | 0.0005 |
| 192 | Anileridine |  | Organ heterocyclic compounds | 0.65 | 1.64 | 0.0002 |
| 193 | 3,4-Dichlorophenylurea |  | --- | 0.65 | 1.63 | 0.0001 |
| 194 | Taurohyodeoxycholic Acid |  | --- | 0.65 | 1.30 | 0.0087 |
| 195 | Methyl 3-Aminopyrazine-2-Carboxylic Acid |  | --- | 0.65 | 1.65 | 0.0002 |
| 196 | Palmitoylcarnitine | C02990 | Lipids and lipid-like molecules | 0.65 | 1.91 | 0.0000 |
| 197 | Alanine | C01401 | Organic acids and derivatives | 0.65 | 1.70 | 0.0003 |
| 198 | Acetylcysteine | C06809 | Organic acids and derivatives | 0.65 | 1.81 | 0.0000 |
| 199 | Gomisin D | C17816 | --- | 0.65 | 1.70 | 0.0003 |
| 200 | D-2-Aminoadipic Acid |  | --- | 0.65 | 1.67 | 0.0005 |
| 201 | Specioside |  | --- | 0.65 | 1.14 | 0.0414 |
| 202 | Diallyl Trisulfide |  | Organosulfur compounds | 0.65 | 1.09 | 0.0166 |
| 203 | Adenosine 5'-Diphospho-Glucose |  | --- | 0.65 | 2.08 | 0.0000 |
| 204 | Palmatine Chloride |  | --- | 0.66 | 1.08 | 0.0416 |
| 205 | Skimmin |  | --- | 0.66 | 1.85 | 0.0000 |
| 206 | Salvianolic Acid C |  | --- | 0.66 | 1.22 | 0.0102 |
| 207 | Tomatine | C10827 | Lipids and lipid-like molecules | 0.67 | 1.07 | 0.0469 |

**Table S4. Identified Significant differences in hippocampal metabolites in the diabetic mice as reversed by D-pinitol.**

| S | MetaboName | KEGGID | Super class | DP/DM | VIP | P value |
| --- | --- | --- | --- | --- | --- | --- |
| 1 | Indole-3-Acetyl-L-Valine |  | --- | 0.55 | 2.08 | 0.0000 |
| 2 | cis-Aconitate | C00417 | Organic acids and derivatives | 0.41 | 1.69 | 0.0002 |
| 3 | Quinidine | C06527 | Alkaloids and derivatives | 0.64 | 1.59 | 0.0004 |
| 4 | DDAO |  | --- | 0.55 | 1.59 | 0.0007 |
| 5 | N,N`-Dimethyl-N,N`-Diphenylurea |  | --- | 0.22 | 1.48 | 0.0027 |
| 6 | Gluconasturtiin | C08417 | Organic oxygen compounds | 0.54 | 1.34 | 0.0059 |
| 7 | Acetaminophen Glucuronide |  | Organic oxygen compounds | 0.31 | 1.22 | 0.0080 |
| 8 | Laccaic Acid A |  | --- | 0.18 | 1.21 | 0.0160 |
| 9 | N-Cyclohexyl-2-Benzothiazol-Amine |  | Organ heterocyclic compounds | 0.34 | 1.18 | 0.0178 |
| 10 | Melatonin | C01598 | Organ heterocyclic compounds | 0.01 | 1.22 | 0.0270 |
| 11 | Veratramine | C10829 | --- | 0.12 | 1.07 | 0.0329 |
| 12 | Arctiin | C16915 | --- | 0.40 | 1.13 | 0.0355 |
| 13 | 2-Methylanthra-9,10-Quinone |  | --- | 1.79 | 1.45 | 0.0012 |
| 14 | Deoxyguanylic Acid | C00362 | Nucleosides, nucleotides, and analogues | 1.75 | 1.29 | 0.0067 |
| 15 | Canthin-6-One | C09098 | --- | 10.48 | 1.21 | 0.0197 |
| 16 | Sarafloxacin HCl |  | --- | 3.11 | 1.61 | 0.0005 |
| 17 | Pinoxaden |  | --- | 1.52 | 1.38 | 0.0049 |
| 18 | Aloenin |  | --- | 2.26 | 1.51 | 0.0014 |
| 19 | Malonic Acid | C00383 | Organic acids and derivatives | 1.82 | 1.95 | 0.0000 |
| 20 | 3-Methylhistidine | C01152 | Organic acids and derivatives | 2.60 | 1.30 | 0.0086 |
| 21 | 2-Hydroxyacetanilide |  | Benzenoids | 3.83 | 1.81 | 0.0000 |
| 22 | Syringic Acid | C10833 | Benzenoids | 2.41 | 1.51 | 0.0012 |
| 23 | Bisanhydrorutilantinone |  | --- | 8.99 | 1.81 | 0.0000 |
| 24 | Tonantzitlolone B |  | --- | 3.29 | 1.64 | 0.0001 |
| 25 | Isodolichantoside |  | --- | 1.51 | 1.23 | 0.0066 |
| 26 | Scytonemin |  | --- | 2.62 | 1.83 | 0.0000 |
| 27 | Protodioscin | C08907 | Lipids and lipid-like molecules | 1.53 | 1.88 | 0.0000 |
| 28 | Fenoldopam | C07693 | Organ heterocyclic compounds | 1.65 | 1.43 | 0.0059 |
| 29 | Mevalonic Acid Lactone |  | Organ heterocyclic compounds | 2.14 | 1.60 | 0.0018 |
| 30 | Anileridine |  | Organ heterocyclic compounds | 1.71 | 1.34 | 0.0068 |
| 31 | Taurohyodeoxycholic Acid |  | --- | 1.66 | 1.15 | 0.0153 |
| 32 | Diallyl Trisulfide |  | Organosulfur compounds | 1.74 | 1.16 | 0.0200 |

**Table S5. Summary KEGG pathway of Significant differences in hippocampal proteins in the diabetic mice.**

| NO | Pathway ID | Pathway name | Identified components | Count | P value |
| --- | --- | --- | --- | --- | --- |
| 1 | mmu04260 | Cardiac muscle contraction | Atp1a1/Atp1b1/Cox4i1/Cox5a/COX2/Cox7a2/Uqcr10/Cox6c/Cox7c/Tpm3/Tpm1/COX1 | 12 | 0.0000 |
| 2 | mmu04714 | Thermogenesis | Atp5o/Ndufa10/Cox4i1/Acsl6/Cox5a/COX2/Ndufa4/Cox7a2/Uqcr10/Atp5k/Cox6c/Ndufc2/Cox7c/Acsl3/Cpt2/Slc25a20/ND2/Rps6ka2/COX1 | 19 | 0.0000 |
| 3 | mmu05012 | Parkinson disease | Tubb4b/Septin5/Atp5o/Ndufa10/Cox4i1/Cox5a/COX2/Ndufa4/Cox7a2/Uqcr10/Cox6c/Ndufc2/Cox7c/Txn2/Slc39a7/Tubb2a/Hspa5/ND2/Gpr37/COX1 | 20 | 0.0000 |
| 4 | mmu05020 | Prion disease | Tubb4b/Atp5o/Ndufa10/Cox4i1/Cox5a/COX2/Ndufa4/Cox7a2/Uqcr10/Cox6c/Ndufc2/Cox7c/Hspa8/Tubb2a/Hspa5/Stip1/Ppp3r1/ND2/COX1/C1qa | 20 | 0.0000 |
| 5 | mmu00190 | Oxidative phosphorylation | Atp5o/Ndufa10/Cox4i1/Cox5a/COX2/Ndufa4/Cox7a2/Uqcr10/Atp5k/Cox6c/Ndufc2/Cox7c/ND2/COX1 | 14 | 0.0000 |
| 6 | mmu05415 | Diabetic cardiomyopathy | Atp5o/Ndufa10/Cox4i1/G6pdx/Cox5a/COX2/Ndufa4/Cox7a2/Uqcr10/Cox6c/Ndufc2/Slc2a1/Cox7c/Cpt2/Mpc1/ND2/COX1 | 17 | 0.0000 |
| 7 | mmu05014 | Amyotrophic lateral sclerosis | Tubb4b/Rab5a/Atp5o/Ndufa10/Cox4i1/Cox5a/COX2/Ndufa4/Cox7a2/Uqcr10/Cox6c/Ndufc2/Cox7c/Ncbp1/Ccs/Tubb2a/Hspa5/Pfn1/Ppp3r1/Pfn2/ND2/Dnal1/COX1 | 23 | 0.0000 |
| 8 | mmu05208 | Chemical carcinogenesis - reactive oxygen species | Atp5o/Ndufa10/Akr1a1/Cox4i1/Cox5a/COX2/Ndufa4/Cox7a2/Uqcr10/Cox6c/Ndufc2/Cox7c/ND2/Gsta4/COX1/Mgst3 | 16 | 0.0000 |
| 9 | mmu05022 | Pathways of neurodegeneration - multiple diseases | Tubb4b/Septin5/Rab5a/Atp5o/Ndufa10/Cox4i1/Cox5a/COX2/Ndufa4/Cox7a2/Uqcr10/Cox6c/Ndufc2/Cox7c/Ccs/Tubb2a/Hspa5/Ppp3r1/Ppid/ND2/Dnal1/Gpr37/COX1 | 23 | 0.0001 |
| 10 | mmu05016 | Huntington disease | Tubb4b/Atp5o/Ndufa10/Cox4i1/Cox5a/COX2/Ndufa4/Cox7a2/Uqcr10/Cox6c/Ndufc2/Cox7c/Hdac2/Tubb2a/ND2/Dnal1/COX1 | 17 | 0.0001 |
| 11 | mmu04141 | Protein processing in endoplasmic reticulum | Erlec1/Ube2d3/Hspa8/Hsp90ab1/Hsp90aa1/Hspa5/Calr/Hsph1/Rrbp1/Pdia6/Sec24c/Cryab | 12 | 0.0002 |
| 12 | mmu04932 | Non-alcoholic fatty liver disease | Ndufa10/Cox4i1/Cox5a/COX2/Ndufa4/Cox7a2/Uqcr10/Cox6c/Ndufc2/Cox7c/COX1 | 11 | 0.0003 |
| 13 | mmu05010 | Alzheimer disease | Tubb4b/Atp5o/Ndufa10/Cox4i1/Cox5a/COX2/Ndufa4/Cox7a2/Uqcr10/Cox6c/Ndufc2/Cox7c/Slc39a7/Tubb2a/Ppp3r1/Ppid/ND2/COX1 | 18 | 0.0008 |
| 14 | mmu04723 | Retrograde endocannabinoid signaling | Gnb2/Ndufa10/Gng3/Ndufa4/Gabrb2/Gng12/Ndufc2/Gng7/Gng13/ND2 | 10 | 0.0009 |
| 15 | mmu03040 | Spliceosome | Acin1/Ncbp1/Lsm3/Cwc15/Hspa8/Ddx39b/Sf3a3/Crnkl1/Snrpb | 9 | 0.0017 |
| 16 | mmu04724 | Glutamatergic synapse | Glul/Gnb2/Gng3/Gng12/Gng7/Shank3/Gng13/Ppp3r1 | 8 | 0.0022 |
| 17 | mmu04727 | GABAergic synapse | Glul/Gnb2/Gng3/Gabrb2/Gng12/Gng7/Gng13 | 7 | 0.0023 |
| 18 | mmu00062 | Fatty acid elongation | Hadha/Acot7/Acot2/Them4 | 4 | 0.0027 |
| 19 | mmu05230 | Central carbon metabolism in cancer | Ldhb/G6pdx/Slc2a1/Pfkl/Ntrk3/Kit | 6 | 0.0028 |
| 20 | mmu00071 | Fatty acid degradation | Hadha/Acsl6/Acsl3/Eci2/Cpt2 | 5 | 0.0041 |
| 21 | mmu05132 | Salmonella infection | Tubb4b/Rab5a/Txn2/Vps41/Tubb2a/Hsp90ab1/Hsp90aa1/Arpc2/Pfn1/Pfn2/Arpc5/Fbxo22 | 12 | 0.0058 |
| 22 | mmu04371 | Apelin signaling pathway | Gnb2/Camk4/Gng3/Gng12/Gng7/Rras2/Gng13/Hdac5 | 8 | 0.0070 |
| 23 | mmu01212 | Fatty acid metabolism | Hadha/Acsl6/Acsl3/Cpt2/Fads2 | 5 | 0.0086 |
| 24 | mmu03320 | PPAR signaling pathway | Acsl6/Plin4/Acsl3/Cpt2/Fabp7/Fads2 | 6 | 0.0097 |
| 25 | mmu04964 | Proximal tubule bicarbonate reclamation | Atp1a1/Atp1b1/Mdh1 | 3 | 0.0098 |
| 26 | mmu05032 | Morphine addiction | Gnb2/Gng3/Gabrb2/Gng12/Gng7/Gng13 | 6 | 0.0108 |
| 27 | mmu00620 | Pyruvate metabolism | Mdh1/Ldhb/Akr1a1/Glo1 | 4 | 0.0122 |
| 28 | mmu00650 | Butanoate metabolism | Oxct1/Hadha/Bdh1 | 3 | 0.0190 |
| 29 | mmu04014 | Ras signaling pathway | Gnb2/Rab5a/Gng3/Gng12/Gng7/Rras2/Gng13/Rac3/Kit/Rasa1 | 10 | 0.0225 |
| 30 | mmu04978 | Mineral absorption | Atp1a1/Atp1b1/Fth1/Cybrd1 | 4 | 0.0243 |
| 31 | mmu00640 | Propanoate metabolism | Ldhb/Hadha/Suclg2 | 3 | 0.0249 |
| 32 | mmu05034 | Alcoholism | Gnb2/Camk4/Gng3/H2aw/Gng12/Gng7/Hdac2/Gng13/Hdac5 | 9 | 0.0249 |
| 33 | mmu04725 | Cholinergic synapse | Gnb2/Camk4/Gng3/Gng12/Gng7/Gng13 | 6 | 0.0273 |
| 34 | mmu04217 | Necroptosis | Glul/Fth1/H2aw/Chmp6/Spata2l/Hsp90ab1/Hsp90aa1/Ppid | 8 | 0.0281 |
| 35 | mmu01232 | Nucleotide metabolism | Adsl/Adssl1/Cmpk1/Ak5/Nudt16 | 5 | 0.0287 |
| 36 | mmu00280 | Valine, leucine and isoleucine degradation | Oxct1/Hadha/Hibadh/Auh | 4 | 0.0290 |
| 37 | mmu04130 | SNARE interactions in vesicular transport | Stx1b/Vamp2/Stx16 | 3 | 0.0294 |
| 38 | mmu01040 | Biosynthesis of unsaturated fatty acids | Acot7/Acot2/Fads2 | 3 | 0.0318 |
| 39 | mmu01240 | Biosynthesis of cofactors | Akr1a1/Mthfd1l/Adsl/Pmm1/Adssl1/Cmpk1/Ak5 | 7 | 0.0365 |
| 40 | mmu00051 | Fructose and mannose metabolism | Pmm1/Pfkl/Gmds | 3 | 0.0368 |
| 41 | mmu04612 | Antigen processing and presentation | Hspa8/Hsp90ab1/Hsp90aa1/Hspa5/Calr | 5 | 0.0371 |
| 42 | mmu04114 | Oocyte meiosis | Anapc1/Ywhae/Ywhab/Ywhag/Ppp3r1/Rps6ka2 | 6 | 0.0378 |
| 43 | mmu01250 | Biosynthesis of nucleotide sugars | Pmm1/Galk1/Gmds | 3 | 0.0422 |
| 44 | mmu00250 | Alanine, aspartate and glutamate metabolism | Glul/Adsl/Adssl1 | 3 | 0.0451 |
| 45 | mmu04216 | Ferroptosis | Acsl6/Fth1/Acsl3 | 3 | 0.0480 |

**Table S6. Summary KEGG pathway of Significant differences in hippocampal proteins in the diabetic mice as reversed by D-pinitol.**

| NO | Pathway ID | Pathway name | Identified components | Count | P value |
| --- | --- | --- | --- | --- | --- |
| 1 | mmu05132 | Salmonella infection | Tubb2a/Pfn1/Pfn2/Arpc5/Fbxo22 | 5 | 0.0037 |
| 2 | mmu05014 | Amyotrophic lateral sclerosis | Tubb2a/Pfn1/Pfn2/Dnal1/Cox5a/Cox6c | 6 | 0.0038 |
| 3 | mmu03040 | Spliceosome | Ddx39b/Crnkl1/Snrpb/Lsm3 | 4 | 0.0040 |
| 4 | mmu04015 | Rap1 signaling pathway | Pfn1/Pfn2/Rapgef6/Rap1gap | 4 | 0.0115 |
| 5 | mmu05208 | Chemical carcinogenesis - reactive oxygen species | Gsta4/Mgst3/Cox5a/Cox6c | 4 | 0.0130 |
| 6 | mmu04714 | Thermogenesis | Rps6ka2/Cox5a/Atp5k/Cox6c | 4 | 0.0148 |
| 7 | mmu00190 | Oxidative phosphorylation | Cox5a/Atp5k/Cox6c | 3 | 0.0181 |
| 8 | mmu05012 | Parkinson disease | Tubb2a/Septin5/Cox5a/Cox6c | 4 | 0.0230 |
| 9 | mmu04720 | Long-term potentiation | Rps6ka2/Camk4 | 2 | 0.0312 |
| 10 | mmu04530 | Tight junction | Arpc5/Rapgef6/Dlg1 | 3 | 0.0314 |
| 11 | mmu00982 | Drug metabolism - cytochrome P450 | Gsta4/Mgst3 | 2 | 0.0348 |
| 12 | mmu05016 | Huntington disease | Tubb2a/Dnal1/Cox5a/Cox6c | 4 | 0.0354 |
| 13 | mmu00480 | Glutathione metabolism | Gsta4/Mgst3 | 2 | 0.0357 |
| 14 | mmu00980 | Metabolism of xenobiotics by cytochrome P450 | Gsta4/Mgst3 | 2 | 0.0366 |
| 15 | mmu01524 | Platinum drug resistance | Gsta4/Mgst3 | 2 | 0.0432 |
| 16 | mmu05022 | Pathways of neurodegeneration - multiple diseases | Tubb2a/Dnal1/Septin5/Cox5a/Cox6c | 5 | 0.0435 |
| 17 | mmu05204 | Chemical carcinogenesis - DNA adducts | Gsta4/Mgst3 | 2 | 0.0472 |

**Table S7. Summary KEGG pathway of Significant differences in hippocampal metabolites in the diabetic mice.**

| **NO** | **Pathway ID** | **Pathway name** | **KEGGID** | **Count** | **P value** |
| --- | --- | --- | --- | --- | --- |
| 1 | mmu04977 | Vitamin digestion and absorption | C05443/C00864/C00250/C00255 | 4 | 0.0005 |
| 2 | mmu01230 | Biosynthesis of amino acids | C00019/C00062/C03406/C01005/C00158/C00449 | 6 | 0.0014 |
| 3 | mmu00240 | Pyrimidine metabolism | C00178/C00295/C00105/C00383 | 4 | 0.0035 |
| 4 | mmu00410 | beta-Alanine metabolism | C00864/C00386/C00383 | 3 | 0.0035 |
| 5 | mmu01240 | Biosynthesis of cofactors | C00019/C00275/C00864/C00250/C00295/C00255/C00105/C00130/C00158 | 9 | 0.0036 |
| 6 | mmu00310 | Lysine degradation | C00164/C00487/C00449 | 3 | 0.0124 |
| 7 | mmu00020 | Citrate cycle (TCA cycle) | C00417/C00158 | 2 | 0.0159 |
| 8 | mmu00740 | Riboflavin metabolism | C01727/C00255 | 2 | 0.0159 |
| 9 | mmu00220 | Arginine biosynthesis | C00062/C03406 | 2 | 0.0208 |
| 10 | mmu05323 | Rheumatoid arthritis | C05443 | 1 | 0.0291 |
| 11 | mmu00250 | Alanine, aspartate and glutamate metabolism | C03406/C00158 | 2 | 0.0301 |
| 12 | mmu04742 | Taste transduction | C00130/C00158 | 2 | 0.0385 |
| 13 | mmu04142 | Lysosome | C00275 | 1 | 0.0386 |
| 14 | mmu04150 | mTOR signaling pathway | C00062 | 1 | 0.0386 |
| 15 | mmu02010 | ABC transporters | C00062/C00255/C00487/C00392 | 4 | 0.0445 |

**Table S8. Summary KEGG pathway of Significant differences in hippocampal metabolites in the diabetic mice as reversed by D-pinitol.**

| NO | **Pathway ID** | **Pathway name** | **KEGGID** | **Count** | **P value** |
| --- | --- | --- | --- | --- | --- |
| 1 | mmu04713 | Circadian entrainment | C01598 | 1 | 0.0125 |
| 2 | mmu01210 | 2-Oxocarboxylic acid metabolism | C08417/C00417 | 2 | 0.0134 |
| 3 | mmu00020 | Citrate cycle (TCA cycle) | C00417 | 1 | 0.0276 |
| 4 | mmu00410 | beta-Alanine metabolism | C00383 | 1 | 0.0439 |
