# Supplementary figures and images for: Integrated proteomics and metabolomics analysis of D-pinitol function during hippocampal damage in streptozocin-induced aging-accelerated mice
Source: Front Mol Neurosci. 2023 Oct 30;16:1251513. doi: 10.3389/fnmol.2023.1251513 (PMC10664147; doi:10.3389/fnmol.2023.1251513)

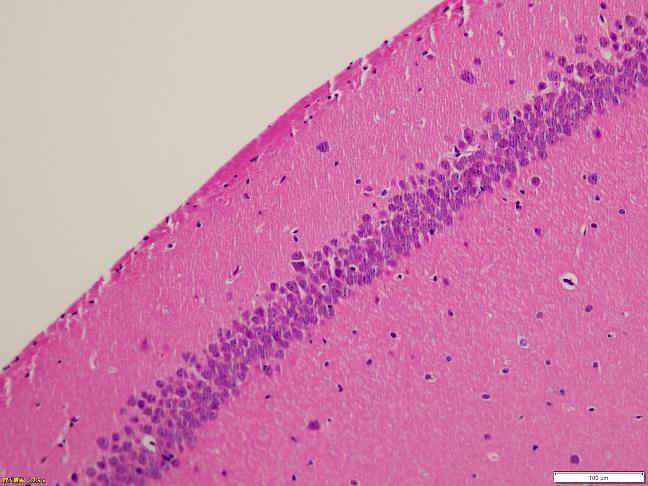

Supplement: Supplementary file 11 [file Image_1.jpg]

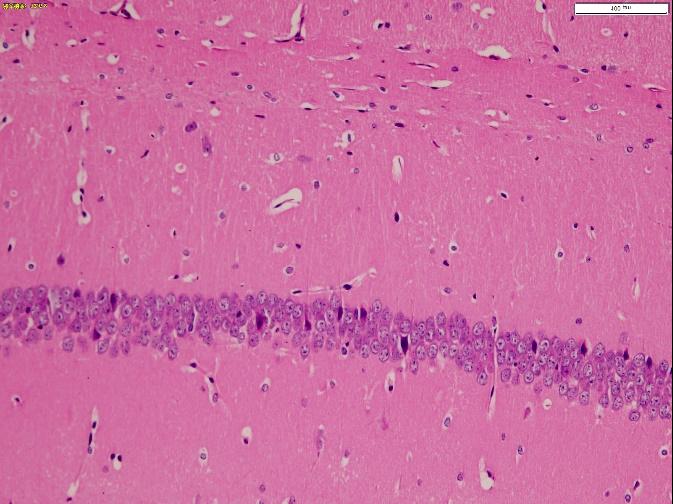

Supplement: Supplementary file 12 [file Image_2.jpg]

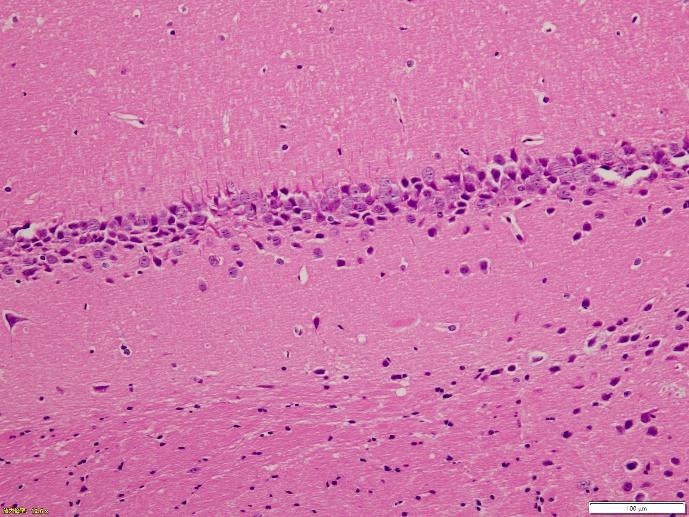

Supplement: Supplementary file 13 [file Image_3.jpg]

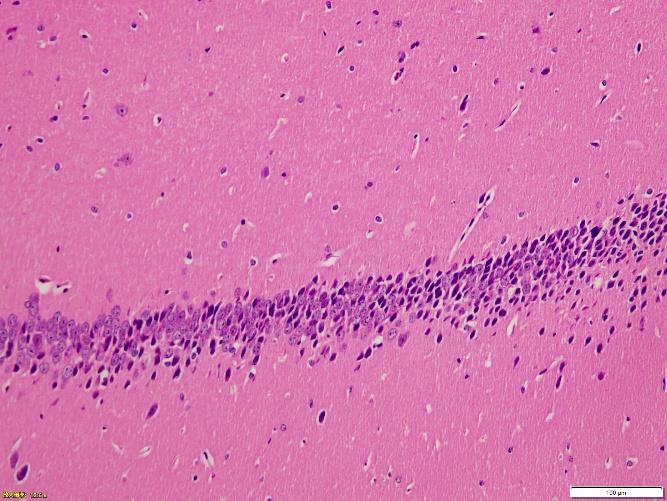

Supplement: Supplementary file 14 [file Image_4.jpg]

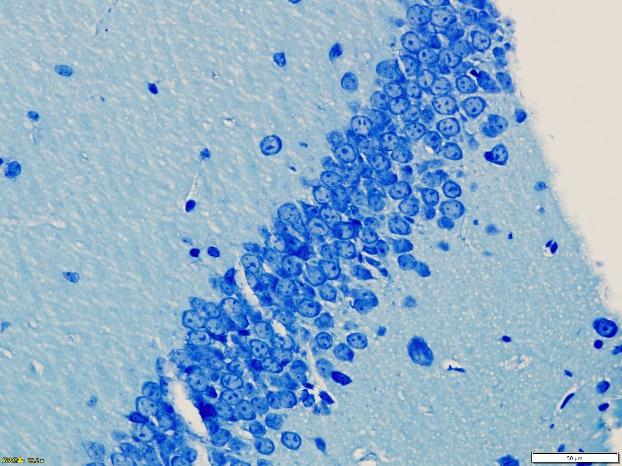

Supplement: Supplementary file 15 [file Image_5.jpg]

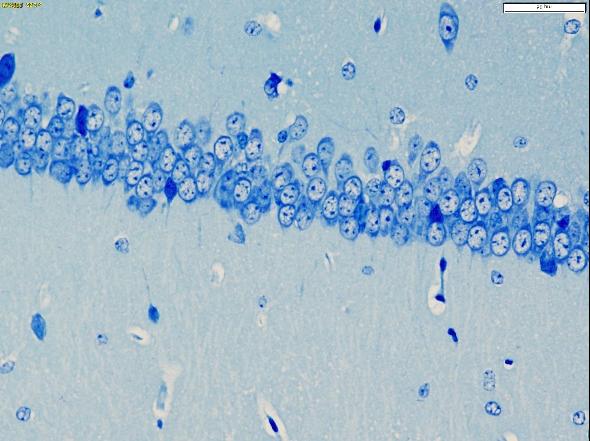

Supplement: Supplementary file 16 [file Image_6.jpg]

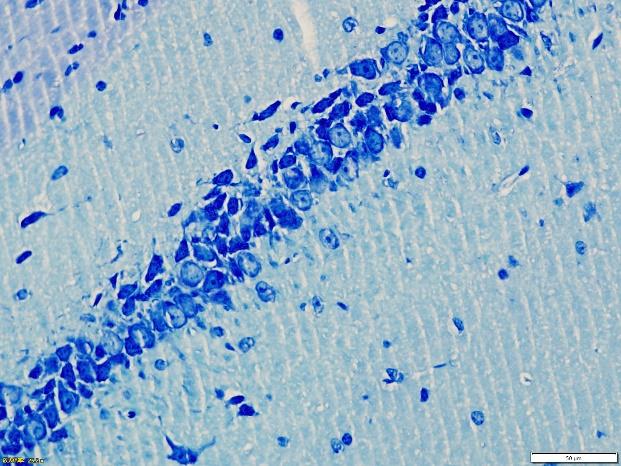

Supplement: Supplementary file 17 [file Image_7.jpg]

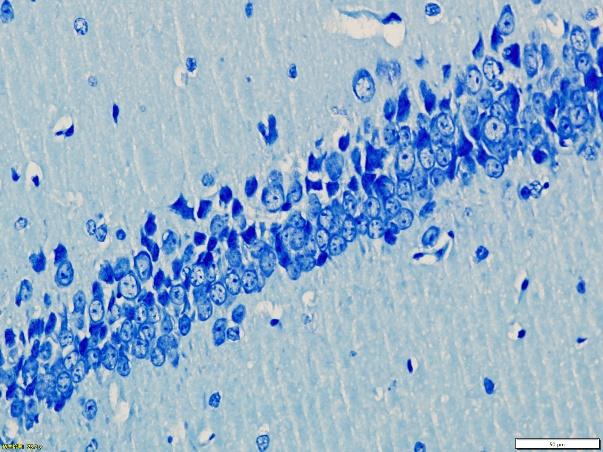

Supplement: Supplementary file 18 [file Image_8.jpg]

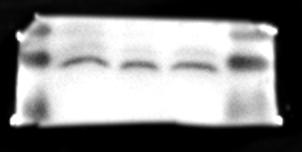

Supplement: Supplementary file 19 [file Image_9.jpg]

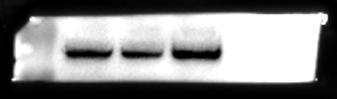

Supplement: Supplementary file 20 [file Image_10.jpg]

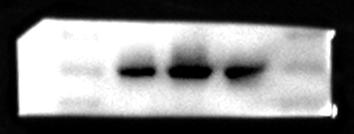

Supplement: Supplementary file 21 [file Image_11.jpg]

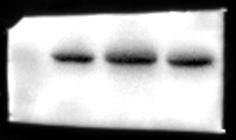

Supplement: Supplementary file 22 [file Image_12.jpg]
